# Using quantitative PCR to identify opportunities to strengthen soil-transmitted helminth control in Solomon Islands: A cross-sectional epidemiological survey

**DOI:** 10.1371/journal.pntd.0010350

**Published:** 2022-05-23

**Authors:** Brandon Le, Naomi Clarke, Sze Fui Hii, Aisling Byrne, Patsy A. Zendejas-Heredia, Susanna Lake, Oliver Sokana, Alam Khattak, Lucia Romani, Daniel Engelman, Titus Nasi, Dickson Boara, John Kaldor, Andrew Steer, Rebecca Traub, Susana Vaz Nery

**Affiliations:** 1 The Kirby Institute, University of New South Wales, Sydney, Australia; 2 The University of Melbourne, Melbourne, Australia; 3 Murdoch Children’s Research Institute, Melbourne, Australia; 4 Ministry of Health & Medical Services, Honiara, Solomon Islands; 5 National Referral Hospital, Honiara, Solomon Islands; 6 Gizo Hospital, Gizo, Solomon Islands; Washington University School of Medicine, UNITED STATES

## Abstract

**Background:**

The Kato-Katz microscopy technique is the global standard for assessment of soil-transmitted helminth (STH) burden. However, major limitations include its poor sensitivity, requirement for rapid sample processing, and inability to differentiate hookworm species nor detect *Strongyloides* spp. infections. We assessed the prevalence and intensity of STH species in Solomon Islands by conducting a province-wide survey using quantitative PCR (qPCR) for diagnosis, which can provide much better characterisation of STH burden than microscopy.

**Methodology/Principal findings:**

We conducted a cross-sectional survey in 18 villages in Western Province to detect infections with six STH species and quantify intensity with three. We used linear mixed model regression to identify potential water, sanitation, and hygiene (WASH) and environmental risk factors for infection. We collected stool specimens from 830 village residents. Overall STH prevalence was 63.3% (range 27.5 to 91.5% across villages), led by *Necator americanus* (54.5% [range 17.5–89.4%]), followed by *Ancylostoma ceylanicum* (15.5% [range 2.8–45.8%]), *Trichuris trichiura* (9.1% [range 0–79.2%]), and *Strongyloides* spp. (3.2% [range 0–29.2%]). Most infections were of light intensity for *N*. *americanus* (85.7%) and *T*. *trichiura* (90.7%). Owning a household latrine was associated with a lower risk of *N*. *americanus* infection (AOR 0.41, 95% CI 0.24–0.68) while greater precipitation was linked to more common *T*. *trichiura* infection (AOR 1.14, 95% CI 1.04–1.25).

**Conclusion/Significance:**

In this first large-scale population survey of STH in the Pacific using qPCR, we found evidence that ivermectin should be incorporated into STH control programmes because of the presence of *T*. *trichiura* and *Strongyloides* spp., both of which are poorly responsive to albendazole. Furthermore, One Health strategies are needed for improved *A*. *ceylanicum* and *Strongyloides* spp. control, WASH access and use should be improved to complement deworming programmes, and control efforts should ideally be expanded to entire communities.

**Trial registration:**

ClinicalTrials.gov Australian and New Zealand Clinical Trials Registry ACTRN12618001086257.

## Author summary

Routine assessments of the burden of intestinal worm infections rely on microscopy-based diagnostic methods, such as the Kato-Katz technique. However major limitations include its poor sensitivity, requirement for rapid sample processing, and inability to differentiate individual hookworm species and detect *Strongyloides* spp. infections. It is important to assess the burden of each of these infections to design control approaches beyond the current core strategy of albendazole preventive chemotherapy. Use of qPCR for diagnosis could address these gaps because it can distinguish all relevant intestinal worm species. We completed a province-wide intestinal worm infections prevalence survey using qPCR in Western Province, Solomon Islands. Overall prevalence was extremely high (62.5%). We identified a high burden of *Necator americanus* (54.5%) and detected *Ancylostoma ceylanicum* (15.5%), *Trichuris trichiura* (9.1%), and *Strongyloides* spp. (3.2%) infections. Increased age and precipitation were associated with higher prevalence of infection, while owning a household latrine was associated with lower odds of *N*. *americanus* infection. Our findings support the need to incorporate ivermectin into deworming programmes for *Strongyloides* spp. and *T*. *trichiura* control as these species are poorly responsive to albendazole, implement One Health strategies to address the zoonotic *A*. *ceylanicum* and *Strongyloides* spp., and expand control efforts to reach entire communities. WASH access should also be improved to complement deworming programmes.

## Introduction

Infections with soil-transmitted helminths (STHs) including *Ascaris lumbricoides*, *Trichuris trichiura*, *Strongyloides stercoralis* and the hookworms *Necator americanus*, *Ancylostoma duodenale* and *Ancylostoma ceylanicum* represent the most frequently occurring of all neglected tropical diseases (NTDs). STHs infect an estimated 895 million people worldwide [1] with a disease burden of 1.9 million disability-adjusted life years [2] disproportionately impacting on remote and rural communities in low and middle income countries.

Decisions on the implementation and cessation of STH control programmes rely on findings from epidemiological surveys that assess infection prevalence and intensity. The Kato-Katz microscopy technique, recommended by the World Health Organization (WHO), has been the mainstay for STH detection in these surveys. Its attraction is relative simplicity and low resource requirements. However, major limitations include its poor sensitivity [3,4], requirement for rapid sample processing to avoid hookworm egg degradation, and inability to differentiate the three hookworm species, nor detect *Strongyloides* spp. infection [4]. As a zoonotic disease, control of *A*. *ceylanicum* requires an intersectoral approach, that incorporates One Health strategies as well as preventive chemotherapy [5] particularly in endemic countries in the Asia-Pacific region where high burdens have been documented [6]. Similar considerations apply to *S*. *stercoralis* also suggested to be a zoonosis [7], and the subject of new targets introduced by WHO to establish a control programme by 2030 [5] which will need to incorporate ivermectin preventive chemotherapy, as albendazole is not effective for this species [8]. Ivermectin also plays an important role in improving *T*. *trichiura* control when used in combination with albendazole [9].

Quantitative real-time polymerase chain reaction (qPCR) is a highly sensitive molecular diagnostic assay that can analyse preserved samples and detect infections with all relevant STH species in stool samples [4]. It has been validated for both STH diagnosis and measuring the infection intensity [3,10,11], and so far primarily used in large-scale surveys in the context of clinical trials [12–15] or evaluation of STH transmission elimination status [16]. In settings with diverse STH species, qPCR is far superior to the Kato-Katz method in its ability to provide the detailed characterisation of STH burden needed to guide interventions.

Surveys in two of the nine Solomon Islands provinces have reported an overall prevalence of up to 52.7%, with hookworm being the most prevalent species [17–19], as determined by copro-microscopy methods [17–21]. These extremely high prevalences may be explained by poor access to water, sanitation, and hygiene (WASH) facilities in many parts of the country, and exposure to environmental conditions that are favourable for STH development and transmission, in the absence of a national STH control programme.

In the context of a trial of two versus one dose(s) of ivermectin administration for scabies, we assessed the prevalence and intensity of STH, including *A*. *ceylanicum*, *Strongyloides* spp., and *T*. *trichiura*, in Western Province of Solomon Islands using qPCR. We also aimed to identify WASH and environmental risk factors associated with infection. To our knowledge, this study is the first qPCR prevalence survey of STH infections in the Pacific region and only the second in the world in any low- or middle-income country [12].

## Methods

### Ethics statement

The survey was embedded within the baseline assessment undertaken for a cluster-randomised control trial of ivermectin mass drug administration (Australian and New Zealand Clinical Trials Registry ACTRN12618001086257), which included impact on STH infections as a secondary outcome. The trial was approved by the Solomon Islands Health Research and Ethics Review Board (HRE005/18) and the Royal Children’s Hospital Human Research Ethics Committee, Melbourne, Australia (38099A).

Written informed consent was provided prior to data collection, including obtaining signed consent from all adults and parents or guardians of children under 18 years old. All residents aged 12 months or more in the study villages were eligible to participate.

### Study design and participants

This study took place in Western Province of Solomon Islands. Solomon Islands is an archipelago situated in the Western Pacific region consisting of approximately 695,000 people [22]. Western province is one of nine provinces in the country and is the third most populous with an estimated population of 76,649 people [22]. We conducted a cross-sectional survey of residents of 18 villages in Western Province between May and July 2019. Details of the trial design can be found in the published trial protocol [23]. The 20 villages for the trial were selected randomly from all those in the province with a population size of between 180 to 300 people. For logistical reasons, the STH survey could not be conducted in the first two villages.

### Specimen collection

A local health promotion team conducted community awareness visits approximately one month prior to the commencement of the study. One day prior to stool collection, trained field team members visited villages to seek permission from village leaders to conduct research. Following approval, the team informed residents of the study either through household visits or community meetings at a central location, provided verbal instructions on how to provide a stool sample and offered the opportunity to ask questions. Each household member was given a stool collection kit containing a 70 ml plastic faeces specimen jar, gloves, a study information sheet, and written instructions on providing a sample. Residents were asked to self-collect a fresh stool sample the following day and drop it off the same day to a central location, ensuring they did so before administration of ivermectin, which was also planned to take place that day. Participants were also asked to participate in a WASH questionnaire administered by local field staff after samples were dropped off. A single aliquot of stool (3g) per participant was fixed in 5% w/v potassium dichromate upon receipt and kept at room temperature for at least 4 weeks due to limited access to electricity and a refrigerator in the field, until reaching the University of Melbourne (UoM) lab where they were kept at at 4°C until DNA extraction. This resulted in embryonation of STH eggs, which was confirmed with microscopic examination of a random subset of positive samples at the UoM.

### Quantitative PCR analysis

All samples were couriered to the University of Melbourne for qPCR analysis. Genomic DNA was extracted from a single 200 mg aliquot of each stool sample using a Maxwell RSC Pure Food GMO and Authentication Kit, Promega (Promega Corporation, US) as per manufacturer’s instructions, with the following modifications: an additional bead-beating step with 400μl CTAB Buffer using a FastPrep-24 5G Instrument (MP Biomedicals) and 0.5mm Zirconia/Silica beads (Daintree Scientific, AUS). The full DNA extraction and bead-beating protocol was published elsewhere [3]. Following DNA extraction, samples underwent two Taq Man probe-based quadraplex real-time qPCR assays in duplicate to diagnose STH infections with six species (*N*. *americanus*, *A*. *ceylanicum*, *A*. *duodenale*, *T*. *trichiura*, *Strongyloides* spp., *A*. *lumbricoides*) and quantify intensity of infection with three species (*N*. *americanus*, *T*. *trichiura*, *A*. *lumbricoides*) [3,11,24,25]. The first assay was performed to enumerate *A*. *lumbricoides* and *T*. *trichiura* and detect *Strongyloides* spp. using EHV-4 as an internal qPCR control; and the second was performed to enumerate *N*. *americanus*, *A*. *duodenale*, and *A*. *ceylanicum*, using human 16S mitochondrial rRNA as an internal qPCR and DNA extraction control. Nuclease-free water was used as negative control. The sequences of primers and probes and PCR conditions were based on published information [3,10,11,24–30] and are summarised in Table A in S1 Text. The cycling conditions for both assays consisted of the following parameters: denaturation at 95°C for 2 min, followed by 40 cycles of 15 sec at 95°C and annealing at 60°C for 1 min, with no extension phase. All samples with Ct values in duplicate were deemed positive. The full qPCR protocol was published elsewhere [3].

To convert qPCR-derived cycle threshold (Ct) values into eggs per gram (epg) of stool, we used the following linear regression equations derived for embryonated eggs using methods described previously [3]: *A*. *lumbricoides* epg = 10^((Ct-30.048)/-3.2804)^; *T*. *trichiura* epg = 10^((Ct-31.888)/-4.048)^; *N*. *americanus* epg = 10^((Ct-32.657)/-3.878)^. Briefly, these conversion equations were produced based on faecal seeding experiments where parasite-free human faeces were spiked with a serial dilution of known quantities of eggs purified from human and pig faeces that were allowed to embryonate in a 28°C incubator for at least 4 weeks to mirror the embryonated state of the field samples [3]. Water was also added to the incubator to mirror the hot and humid conditions of a tropical setting. Triplicate qPCR assays were performed on the spiked samples then Log10 transformations of original epg were plotted against Ct values to produce linear regression equations that predicted egg count from Ct values for each species [3]. The epg values estimated from the conversion formulae were classified into one of three infection intensity classes (light, moderate, heavy) according to WHO recommended thresholds [31]. Conversion equations were not produced for *A*. *ceylanicum* as we were unable to obtain eggs needed for the seeding experiments.

### Demographic, WASH, and environmental data collection

Demographic and WASH data were collected through an individual, self-reported questionnaire. The WASH section consisted of 12 questions that assessed access to water sources and sanitation facilities, and hygiene behaviours, at the individual and household level.

Global Positioning System coordinates were collected at the village level. Environmental variables were obtained through publicly available sources reporting remotely-sensed data including temperature, precipitation, elevation, soil composition, vegetation, and landcover type geo-referenced at the village level. A summary of the environmental variables used in this analysis, their spatial resolution, temporal resolution, temporal extent, and source are provided in Table 1.

**Table 1:** Environmental variables extracted for the current analysis.

Environmental variable	Parameter	Spatial resolution	Temporal resolution and extent	Variable source
**Ambient temperature** (°C)	Annual mean temperature Maximum temperature of warmest month Minimum temperature of coldest month Mean temperature of wettest quarter Mean temperature of driest quarter Mean temperature of warmest quarter Mean temperature of coldest quarter	1 km	Monthly averages from 1970–2000	Worldclim^1^
**Precipitation** (cm)	Monthly total precipitation Annual total precipitation Total precipitation of wettest Total precipitation of driest month Total precipitation of wettest quarter Total precipitation of driest quarter Total precipitation of warmest quarter Total precipitation of coldest quarter	1 km	Monthly total averages from 1970–2000	Worldclim^1^
**Elevation** (metres)	Mean elevation in metres	30 m	March 2000	NASA ASTER on Terra satellite^2^
**Slope** (degrees)	Mean slope in degrees	30 m	March 2000	NASA ASTER on Terra satellite^2^
**Vegetation** (NDVI, EVI)	Mean vegetation as measured by NDVI & EVI	250 m	16-day averages across 2019	NASA MODIS on Terra satellite^3^
**Landcover** (Using IGBP land classification scheme)	Most occurring land cover type in village	500 m	Yearly average from 2018	NASA MODIS on Terra & Aqua satellites^4^
**Soil pH**	Mean soil water pH on the top 5cm of the soil	250 m	Yearly averages from1901–2016	International Soil Reference & Information Centre (ISRIC) Soilgrids^5^
**Soil texture**	Proportion of soil clay, silt, and sand content on the top 5cm of the soil	250 m	Yearly averages from 1901–2016	ISRIC Soilgrids^5^

^1^Worldclim Version 2.0,

^2^Data package ASTGTM Version 3,

^3^Data package MOD13Q1 Version 6,

^4^Data package MCD12Q1 Version 6,

^5^SoilGrids250m Version 2.0

Environmental data were processed and extracted using QGIS version 3.10 (Open Source Geospatial Foundation Project, Chicago). Specified spatial zones (buffers) were individually shaped, sized, and positioned to capture the entirety of each village. The mean of the raster cells (matrix of pixels containing data) within buffer zones were extracted for the variables temperature, precipitation, elevation, vegetation (Normalised Difference Vegetation Index [NDVI], Enhanced Vegetation Index [EVI]), and soil pH. Slope data were extrapolated from a digital elevation model in QGIS. For landcover, the modal raster cell value was used to classify villages according to the International Geosphere-Biosphere Programme classification system [32] Soil texture was classified using the United States Department of Agriculture system [33] based on the relative proportions of clay, sand, and silt present on the top 5 cm. Soil pH had a narrow range of pH (5.20–5.50) so was dichotomised into 2 classes based on median (5.30).

### Statistical analysis

To take account of the cluster sampling by village, mixed-effects generalised linear models were used to estimate infection prevalence for each species and to examine differences by sex and age. Post-hoc power analysis indicated that a sample size of 810 was sufficient to detect 52.7% prevalence with any STH (derived from the most recent survey [20]) across 18 clusters with 90% power, 5% margin of error, and design effect of 3.0 to account for cluster sampling.

We used the mixed effects methods to also examine WASH and environmental risk factors associated with infection for each species. The model building procedure was based on previous risk factor analyses [12,34]. A series of univariable regressions were first completed for each variable, with variables being retained for the next step of the analysis if their *p* value was less than 0.20 on the Wald test. Retained variables were grouped into theoretically relevant domains with each then subjected to a series of multivariable regressions with sex and age group entered as covariates, and variables retained if their *p* value was less than 0.10 on the Wald test. They were then tested for multicollinearity using variance inflation factors (VIFs), with VIF>5 being used as the criterion for violating collinearity. Issues with collinearity were resolved using the Akaike Information Criterion (AIC) wherein variables with lower AICs were retained as this indicated greater predictive performance. Finally, a backward stepwise elimination variable selection procedure was used until all variables in the model (except sex and age group) had *p* values of less than 0.05 on the Wald test. All base models contained sex and age group as covariates, and household and village as random effect terms. Adjusted odds ratios (AORs) and incidence rate ratios (IRRs) derived from final models and their 95% confidence intervals (CIs) are reported herein. The significance level for final models was set at *p*<0.05. Analyses were completed using Stata version 14.2 (Stata Corporation, College Station, Texas).

## Results

The 18 study villages had an estimated total population of 4920 based on information provided by village leaders. A total of 2392 stool collection kits were distributed, covering 48.6% of the population. Overall, 830 participants provided a stool sample, corresponding to a 34.7% response rate and 16.9% coverage of the population. Of those who provided a sample, 73.5% (n = 619) also completed the WASH questionnaire.

Among the 830 participants, 57.6% were female and the mean age was 21.7 years (SD 20.5). Responses to the WASH questionnaire indicated that 93.5% of participants practiced open defecation, 20.4% lived in dwellings with a household latrine, 67.9% had access to drinking water from an improved water source, and 15.7% always wore shoes outside. The mean annual temperature was 26.9°C (range 26.6–27.2 across villages) and annual precipitation 341.8 cm (range 321.8–353.7). Soil pH levels were in the range 5.20 to 5.30 for 9 villages comprising 41.7% of participants whereas the remaining 58.3% were from villages that had soil pH in the range 5.30 to 5.50. A full summary of descriptive statistics for WASH and environmental variables is provided in Tables B and C in S1 Text.

### STH infection prevalence and intensity

**Fig 1:**
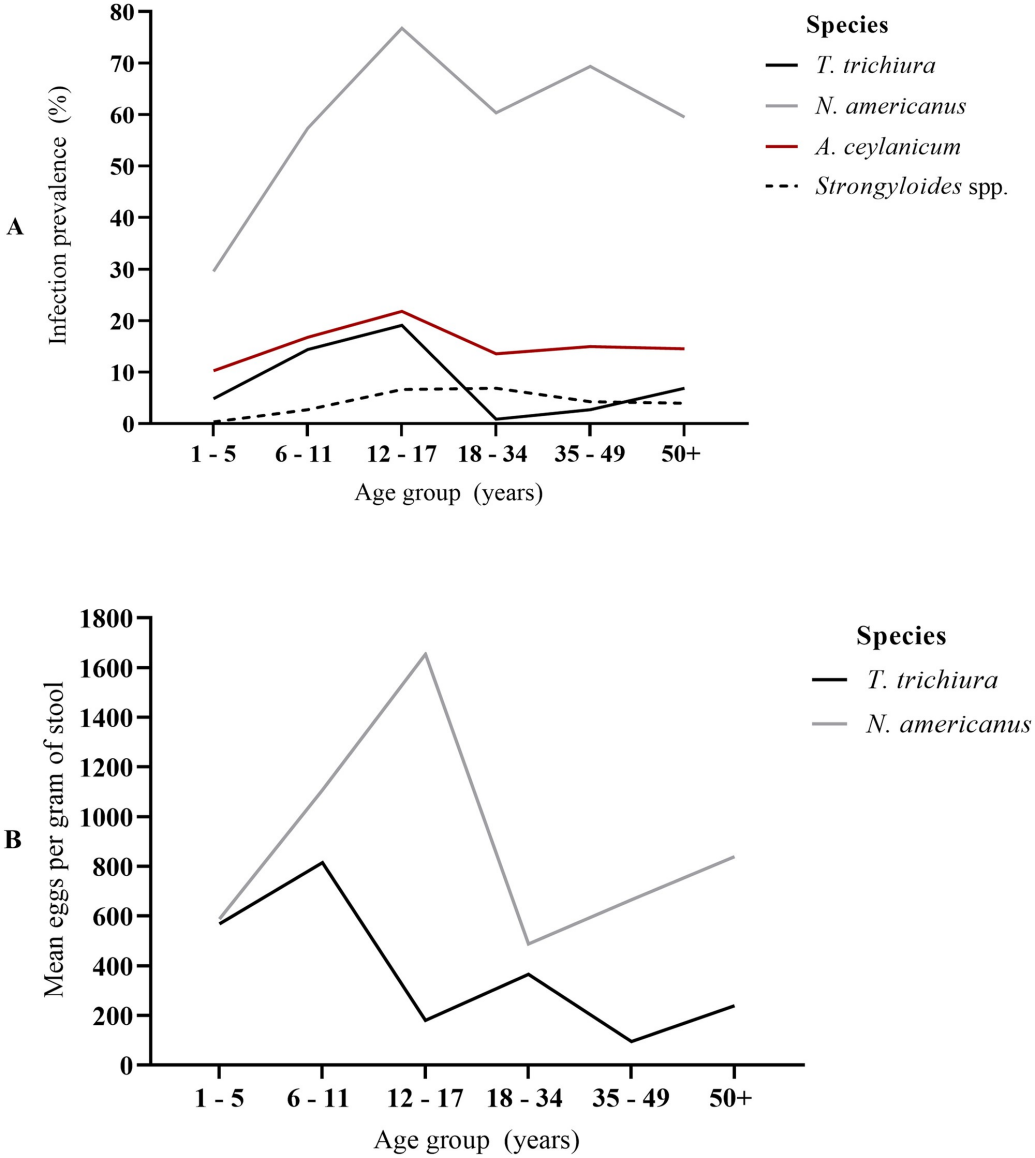
Age- infection profiles by STH species. (A) Prevalence, (B) Intensity as measured by mean eggs per gram of stool. Exact values including 95% confidence intervals and standard deviations are shown in Table 2 (prevalence) and Table E in S1 Text (intensity). *A*. *lumbricoides* infections excluded due to few positive cases.

**Fig 2:**
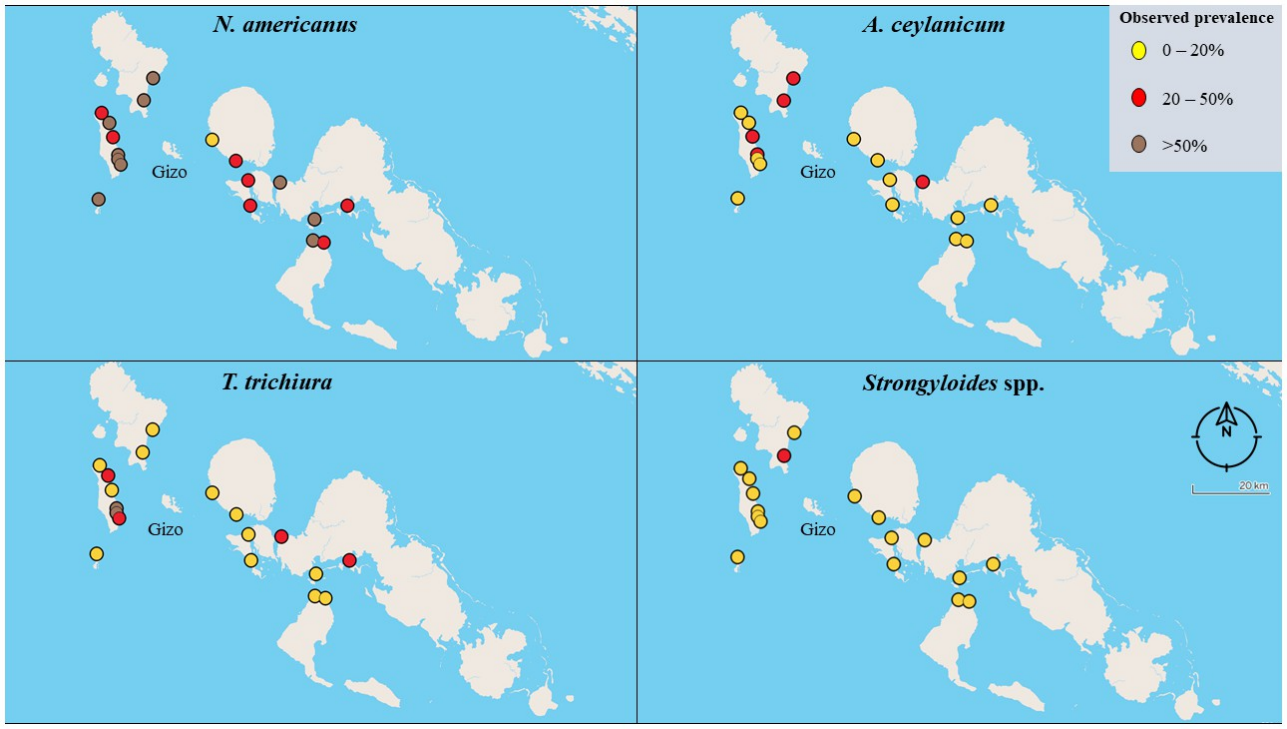
Map showing the observed prevalence of infections with *N*. *americanus*, *A*. *ceylanicum*, *T*. *trichiura*, and *Strongyloides* spp. across 18 villages in Western Province.

The overall prevalence of infection with any STH was 63.3%, ranging from 27.5 to 91.5% across villages (see Tables 2 and 3 and Fig 1). Most common were infections with *N*. *americanus* (54.5%, range 0–79.2%), followed by *A*. *ceylanicum* (15.5%, 2.8–45.8%), *T*. *trichiura* (9.0%, 0–79.2%]) and *Strongyloides* spp. (3.2% [range 0–29.2%]). *A*. *lumbricoides* infection was rare, at 0.08% (range 0–16.7%). Relative to those who were infected, most infections were of light intensity for *A*. *lumbricoides* (66.7%), *T*. *trichiura* (90.7%), and *N*. *americanus* (85.7%). Relative to the entire sample, the prevalence of moderate-to-heavy intensity infections were as follows: *A*. *lumbricoides* (0.2%), *T*. trichiura (1.9%), and *N*. *americanus* (7.6%). Regarding the geographic distribution of infections for each species, we observed that the prevalence of *N*. *americanus* infections was above 20% in 94.4% of villages. A total of 33.3% of villages had *T*. *trichiura* prevalence of above 20%, 27.8% of villages for *A*. *ceylanicum*, and 5.6% of villages for *Strongyloides* spp. (see map, Fig 2).

**Table 2:** Cluster-adjusted STH prevalence by species, stratified by sex and age group.

	Study sample n (%)	Any STH % (95% CI)	*A*. *lumbricoides* % (95% CI)	*T*. *trichiura* % (95% CI)	*N*. *americanus* % (95% CI)	*A*. *ceylanicum* % (95% CI)	A. duodenale % (95% CI)	*Strongyloides* spp. % (95% CI)
**Sex**								
**Male**	352 (42.41)	66.07 (53.69–78.46)	0.09 (0–0.49)	8.25 (0–16.95)	58.50 (45.29–71.71)	13.87 (9.23–18.50)	0	4.27 (0.47–8.06)
**Female**	478 (57.59)	61.37 (49.17–73.57)	0.10 (0–0.51)	8.02 (0.30–16.00)	51.64 (39.89–63.39)	16.49 (10.91–22.06)	0	2.87 (0.18–5.57)
**Age (years)**								
**1–5**	212 (25.54)	41.31 (28.55–54.08)	0.12 (0–0.74)	4.83 (0–11.71)	29.53 (18.68–40.37)	10.27 (4.61–15.94)	0	0.31 (0–1.45)
**6–11**	201 (24.22)	69.75 (51.33–88.18)	0.50 (0–1.47)	14.36 (0–29.61)	57.34 (37.36–77.32)	16.74 (9.72–23.76)	0	2.68 (0–6.25)
**12–17**	89 (10.72)	87.85 (69.60–100)	2.24 (0–6.75)	19.07 (0–45.81)	76.77 (57.71–95.82)	21.77 (7.18–36.34)	0	6.64 (0–17.06)
**18–34**	99 (11.93)	71.07 (56.39–85.75)	0	0.87 (0–4.31)	60.32 (47.62–73.03)	13.53 (3.50–23.56)	0	6.87 (0–14.92)
**35–49**	108 (13.01)	76.58 (60.81–92.35)	0	2.67 (0–9.41)	69.36 (52.10–86.61)	14.96 (5.96–23.96)	0	4.24 (0–11.04)
**≥50**	121 (14.58)	61.98 (53.33–70.63)	0	6.85 (0.30–13.41)	59.50 (50.76–68.25)	14.51 (5.53–23.49)	0	3.69 (0–9.01)
**Total**	**830**	**63.34** **(52.13–74.55)**	**0.08** **(0–0.36)**	**9.08** **(1.27–16.90)**	**54.51** **(43.37–65.64)**	**15.47** **(10.82–20.11)**	**0**	**3.17** **(0.59–5.76)**

**Table 3:** Infection intensity of *A*. *lumbricoides*, *T*. *trichiura*, and *N*. *americanus* as measured by WHO recommended thresholds and mean eggs per gram (epg) of stool.

		Infection intensity class			EPG	
	Prevalence n (%)	Light^†^^a^ % (95% CI)	Moderate^†^^b^ % (95% CI)	Heavy^†^^c^ % (95% CI)	Mean (SD)	Range
***A*. *lumbricoides***	6 (0.08)	66.67 (14.86–95.81)	16.67 (0.91–81.39)	16.67 (0.91–81.39)	54, 264 (117,180.20)	1–291, 868
***T*. *trichiura***	172 (9.08)	90.70 (85.29–94.25)	9.30 (5.75–14.71)	0	478 (1248)	1–8412
***N*. *americanus***	460 (54.51)	85.65 (82.03–88.64)	8.43 (6.16–11.14)	5.92 (4.06–8.57)	920 (1827)	1–12,456

^**†**^Proportion of the population determined to be infected;

^a^Thresholds for light intensity infections: *A*. *lumbricoides* (1–4999 epg), *T*. *trichiura* (1–999 epg), *N*. *americanus* (1–1999 epg);

^b^Thresholds for moderate intensity infections: *A*. *lumbricoides* (5000–49,999 epg), *T*. *trichiura* (1000–9999 epg), *N*. *americanus* (2000–3999 epg);

^c^Thresholds for heavy intensity infections: *A*. *lumbricoides* (≥50,000 epg), *T*. *trichiura* (≥10,000 epg), *N*. *americanus* (≥4000 epg).

In the analysis of risk factors (Tables 4 and 5), males were more likely to be infected with *N*. *americanus*, than females (AOR 1.63, 95% CI 1.13–2.34, *p* = 0.008), and there was a pattern of increasing risk of infection with age relative to young children aged 1–5 years (6–11 years [AOR 5.16, 95% CI 3.04–8.77, *p*<0.001]; 12–17 years [AOR 9.55, 95% CI 4.63–19.68, *p*<0.001]; 18–34 years [AOR 5.79, 95% CI 3.04–11.01, *p*<0.001]; 35–49 years [AOR 6.91, 95% CI 3.66–13.02, *p*<0.001]; ≥50 years [AOR 6.32, 95% CI 3.43–11.64, *p*<0.001]). For *T*. *trichiura*, there was no detectable difference in infection prevalence by sex but we observed increasing prevalence by age within children (6–11 years; AOR 3.0, 95% CI 1.49–6.01, *p* = 0.002) and adolescents (12–17 years; AOR 5.99, 95% CI 2.56–14.00, *p*<0.001), compared young children (see Table 5). There were no detectable differences in *Strongyloides* spp. infection prevalence by sex, but again higher levels in adolescents (AOR 10.11, 95% CI 2.58–39.60, *p* = 0.001) and adults (18–34 years, AOR 5.08, 95% CI 1.17–22.05) than young children. There was no evidence of differences in prevalence by sex or age for *A*. *ceylanicum*. For infection intensity, there was a pattern of increasing *N*. *americanus* egg counts with age within in adolescents (IRR 4.04, 95% CI 2.11–7.71, *p*<0.001) and older adults (≥50 years; IRR 2.55, 95% CI 1.28–5.06, *p* = 0.007), compared to young children (Table E in S1 Text). There were no detectable differences in *T*. *trichiura* infection intensity by age or sex. Demographic differences in *A*. *lumbricoides* infections were not examined due to few positive cases (n = 6).

**Table 4:** Risk factors associated with *N*. *americanus*, *A*. *ceylanicum*, and undifferentiated hookworm infection.

	*N*. *americanus* (n = 460)			*A*. *ceylanicum* (n = 140)			Hookworm (undifferentiated) (n = 489)		
Risk factor variable	aOR	95% CI	*P* value	aOR	95% CI	*P* value	aOR	95% CI	*P* value
Male sex^a^	1.63	1.13–2.34	0.008	0.77	0.51–1.16	0.212	1.59	1.11–2.26	<0.001
Age group 6–11 years^b^	5.16	3.04–8.77	<0.001	1.78	0.99–3.20	0.084	4.05	2.45–6.70	<0.001
Age group 12–17 years^b^	9.55	4.63–19.68	<0.001	2.77	1.39–5.51	0.004	7.12	3.54–14.34	<0.001
Age group 18–34 years^b^	5.79	3.04–11.01	<0.001	1.34	0.65–2.76	0.435	5.09	2.72–9.51	<0.001
Age group 35–49 years^b^	6.91	3.66–13.02	<0.001	1.31	0.65–2.65	0.449	5.39	2.93–9.94	<0.001
Age group ≥50 years^b^	6.32	3.43–11.64	<0.001	1.34	0.67–2.66	0.405	4.31	2.43–7.64	<0.001
*N*. *americanus* co-infection	-	-	-	4.58	2.58–8.14	<0.001	-	-	-
*A*. *ceylanicum* co-infection	4.36	2.36–8.06	<0.001	-	-	-	-	-	-
*T*. *trichiura* co-infection	2.79	1.39–5.60	0.004	-	-	-	3.36	1.64–6.89	0.001
*Strongyloides* spp. co-infection	-	-	-	3.19	1.39–7.34	0.006	-	-	-
Main drinking water is from improved water source^c^*	-	-	-	2.71	1.41–5.24	0.003	-	-	-
Has toilet/latrine in household	0.41	0.24–0.68	<0.001	-	-	-	0.45	0.27–0.74	0.002
Soil pH (pH >5.30–5.50)^d^	2.92	1.29–6.60	0.010	-	-	-	2.85	1.27–6.37	0.011

“95% CI” denotes 95% confidence interval, “aOR” denotes adjusted odds ratio.

These results were derived from a model building procedure where variables were removed from the analysis if they did not meet the criterion *p* value at each stage of the analysis. Variables with blank cells indicate that these were removed in an earlier stage. Tables F-K in S1 Text summarises the *p* values associated with each variable at each stage of the model building procedure.

**Reference categories**:

^a^Female sex;

^b^Age group 1–5 years;

^c^Main drinking water is from unimproved source;

^d^Soil pH 5.20–5.30.

*Responses were collapsed into 2 response options in accordance with the WHO and United Nations International Children’s Emergency Fund (UNICEF) Joint Monitoring Programme (JMP) for Water Supply and Sanitation definitions of “improved” (public tap/standpipe, protected spring, rainwater) and “unimproved” (unprotected spring, unprotected well) drinking water sources. Sex and age group were entered as covariates in the model.

**Table 5:** Risk factors associated with *T*. *trichiura*, *Strongyloides* spp., and undifferentiated STH infection.

	*T*. *trichiura* (n = 172)			*Strongyloides* spp. (n = 49)			Any STH (n = 519)		
Risk factor variables	aOR	95% CI	*P* value	aOR	95% CI	*P* value	aOR	95% CI	*P* value
Male sex^a^	1.12	0.68–1.85	0.643	1.86	0.86 0 4.03	0.098	1.49	1.03–2.17	0.033
Age group 6–11 years^b^	3.00	1.49–6.01	0.002	2.57	0.68–9.69	0.162	4.04	2.39–6.83	<0.001
Age group 12–17 years^b^	5.99	2.56–14.00	<0.001	10.11	2.58–36.60	0.001	6.82	3.24–14.34	<0.001
Age group 18–34 years^b^	1.31	0.56–3.09	0.530	5.08	1.17–22.05	0.030	4.73	2.47–9.08	<0.001
Age group 35–49 years^b^	0.98	0.43–2.23	0.960	4.00	0.95–16.90	0.059	5.31	2.77–10.15	<0.001
Age group ≥50 years^b^	0.84	0.31–2.26	0.729	4.00	0.87–18.31	0.074	3.60	2.01–6.44	<0.001
*N*. *americanus* co-infection	3.39	1.66–6.91	<0.001	4.42	1.32–14.75	0.013	-	-	-
*A*. *ceylanicum* co-infection	-	-	-	-	-	-	-	-	-
*T*. *trichiura* co-infection	-	-	-	3.00	1.18–7.62	0.026	-	-	-
*Strongyloides* spp. co-infection	-	-	-	-	-	-	-	-	-
***Wears shoes outside*** ^c^									
Sometimes	0.86	0.41–1.79	0.686	-	-	-	-	-	-
Always	0.19	0.05–0.71	0.013	-	-	-	-	-	-
Has toilet/latrine in household	-	-	-	-	-	-	0.40	0.24–0.68	0.001
Annual precipitation (cm)	1.14	1.04–1.25	0.008	-	-	-	-	-	-
Soil pH (pH >5.30–5.50)^d^	-	-	-	7.47	1.90–29.33	0.004	3.91	1.71–8.97	0.001

**“95% CI” denotes 95% confidence interval, “aOR” denotes adjusted odds ratio**. These results were derived from a model building procedure where variables were removed from the analysis if they did not meet the criterion *p* value at each stage of the analysis. Variables with blank cells indicate that these were removed in an earlier stage. Tables F-K in S1 Text summarises the *p* values associated with each variable at each stage of the model building procedure.

**Reference categories**:

^a^Female sex;

^b^Age group 1–5 years;

^c^Never wears shoes outside;

^d^Soil pH 5.20–5.30.

Sex and age group were entered as covariates in the model.

### WASH and environmental risk factors

As shown in Table 4, participants who reported owning a household toilet/latrine had lower odds of infection with *N*. *americanus* than those who reported not owning a toilet (AOR 0.41, 95% CI 0.24–0.68, *p*<0.001). Participants from villages with less acidic soil were 3 times more likely to have an infection than those from villages with more acidic soil (AOR 2.92, 95% CI 1.29–6.60, *p* = 0.010). Co-infection with *A*. *ceylanicum* (AOR 4.36, 95% CI 2.36–8.06, *p*<0.001) and *T*. *trichiura* (AOR 2.79, 95% CI 1.39–5.60, *p* = 0.004) were associated with a higher odds of *N*. *americanus* infection.

Participants whose main drinking water source was from an improved source had higher odds of *A*. *ceylanicum* infection than those whose water was from an unimproved source (AOR 2.71, 95% CI 1.41–5.24, *p* = 0.003). Co-infection with *N*. *americanus* (AOR 4.58, 95% CI 2.58–8.14, *p*<0.001) and *Strongyloides* spp. (AOR 3.19, 95% CI 1.39–7.34, *p* = 0.006) were associated with a higher prevalence of *A*. *ceylanicum* infection.

Participants who reported always wearing shoes outside were significantly less likely to have *T*. *trichiura* infection (AOR 0.19, 95% CI 0.05–0.71, *p* = 0.013). Greater annual precipitation was associated with marginally higher odds of infection (AOR 1.14, 95% CI 1.04–1.25, *p* = 0.008), as was co-infection with *N*. *americanus* (AOR 3.39, 95% CI 1.66–6.91, *p* = 0.001).

We did not detect any statistically significant associations between WASH variables and *Strongyloides* spp. infection (Table 5). Participants from villages with less acidic soil were 7 times more likely to have *Strongyloides* spp. infections than those from villages with more acidic soil (AOR 7.47, 95% CI 1.90–29.33, *p* = 0.004).

## Discussion

In this study, to our knowledge, we completed the first STH prevalence survey in a low- or middle-income country of the Pacific region that used qPCR, enabling the assessment of *Strongyloides* spp. and individual hookworm species. Previous large-scale STH epidemiological surveys to use qPCR as a stand-alone diagnostic tool include three conducted in Timor-Leste in the context of trials [12–15] and one in Japan to confirm STH transmission elimination [16]. We found that the overall STH prevalence (63.3%) is the highest reported in the country, with previous microscopy-based studies documenting prevalence of up to 52.7% [17–21]. Consistent with two previous surveys using microscopy [17,18], the leading species present was hookworm. *T*. *trichiura*, *Strongyloides* spp. and the zoonotic *A*. *ceylanicum* were also abundant. Most infections were of light intensity for *N*. *americanus*, *T*. *trichiura*, and *A*. *lumbricoides*.

Solomon Islands follows WHO recommendations for STH control wherein school-based albendazole preventive chemotherapy is provided, so far only within the capital city, Honiara. Guidelines specify treatment to groups at highest risk of morbidity, including pre-school and school-aged children, in communities with prevalence above 20%, with the aim of reducing the prevalence of moderate-to-heavy intensity infections to less than 2% [5]. Our findings indicate that STH burden in Solomon Islands is well above these thresholds, highlighting an urgent need to expand deworming to reach all provinces.

However, our findings also show that the drug-based treatment approach alone is unlikely to control STHs, for several reasons. *A*. *ceylanicum* is a zoonoses and is a predominant hookworm of domestic dogs and cats in the Asia Pacific region [35]. *Strongyloides* spp., for which WHO recently introduced control targets to be attained by 2030 [5], is also a potential zoonosis comprising two distinct genetic clades, one restricted to dogs and another infecting humans, non-human primates, dogs and cats [7,36]. Zoonotic transmission therefore needs to be addressed through One Health strategies. Canine and feline population control through desexing programs and treating community dogs and cats with macrocyclic lactone based anthelmintics have been proposed as potentially effective control measures [6,37]. Another barrier is the limited efficacy of albendazole against both *Strongyloides* spp. [8] and *T*. *trichiura* [9]. The co-administration of albendazole and ivermectin has superior efficacy for *T*. *trichiura* [9] and ivermectin monotherapy is highly effective against *S*. *stercoralis* [8]. STH control in Solomon Islands provinces where these species are endemic would therefore be strengthened through the co-administration of albendazole and ivermectin, a strategy that would also provide integrated control of other co-endemic NTDs, including scabies [38].

It is important to note that risk of infection for *N*. *americanus*, *T*. *trichiura*, and *Strongyloides* spp., and greater *N*. *americanus* infection intensity, increased with age. Although school-based treatment programs will benefit children, the considerable adult reservoir in the population suggests that treatment should ideally be expanded to entire communities, perhaps integrating with mass drug administration programmes targeting other NTDs.

Overall, only few WASH and environmental factors emerged as significant predictors of STH infection, possibly due to the presence of homogenously poor WASH access/conditions and limited variation in the environmental data across a small geographical area. We found that owning a household latrine was associated with a lower prevalence of *N*. *americanus* infections, probably because of reduced exposure to contaminated faecal matter [39]. Higher annual precipitation was associated with marginally increased odds of *T*. *trichiura* infection, likely due to moist soil conditions favourable to survival and development of STH eggs and larvae [12]. Precipitation has emerged as a risk factor for other species [12,34]. We found that less acidic soil was associated with a higher risk of infection with *N*. *americanus* and *Strongyloides* spp. consistent with in-vitro findings on the optimal pH being 6.0 [40]. Given some evidence suggesting that STH eggs cannot survive in highly alkaloid conditions (pH>12) [41], the use of lime (a common agricultural practice to improve crop yields) has been suggested as a potential tool for STH control [34], although this hypothesis has not been tested.

An unexpected finding was that individuals who reported always wearing shoes outside had reduced odds of *T*. *trichiura* infection. While shoe-wearing can confer protection against species that enter by percutaneous penetration, *T*. *trichiura* transmission occurs via the faecal-oral route. More likely, increased shoe-wearing behaviour might reflect higher socioeconomic status, which may be a proxy of an unmeasured variable that protects against *T*. *trichiura* exposure. Surprisingly, participants whose drinking water was from an improved source had increased odds of infection with *A*. *ceylanicum*. This might be due to increased exposure to *A*. *ceylanicum* larvae near improved water sources deposited by infected dogs in communities. We did not collect data on STH burden in dogs or contamination of the environment with dog faeces, a key gap for future research to address.

Limitations of this study should be considered. The WHO recommended thresholds for defining infection intensity were created using infection intensity measurements derived from the Kato-Katz technique. While there have been studies, including this one, deriving epg from Ct values [3,10], additional studies are needed to further validate this approach and standardize the assessment of infection intensity using qPCR. There are also several important practical issues to be considered when deciding whether qPCR should be used, given its perceived higher cost and need for specialised equipment and trained personnel when compared to microscopy [42]. Several strategies to address these issues have been proposed, such as sample pooling [43], production of PCR equipment in low- and middle-income countries [44], and transfer of technology including training personnel [44]. Moreover, while we opted to use logistic regression for our environmental analysis, which allows us to adjust for clustering effects, this may have limited our ability to identify environmental risk factors given that regression-based techniques do not allow intercorrelated predictor variables to be included in the same model. Recent evidence suggests that alternative statistical approaches, such as Bayesian networks, may better identify risk factors when predictors are intercorrelated [45].

In conclusion, by using qPCR in a province-wide epidemiological survey in Solomon Islands, we were able to comprehensively assess the burden of all STH species including *Strongyloides* spp. and individual hookworm species. This enabled us to identify opportunities to strengthen STH control in Solomon Islands, particularly incorporating ivermectin preventive chemotherapy into deworming programmes, adopting a One Health framework, and expanding STH control to entire communities. Improving WASH use and access could complement deworming programmes by protecting against exposure pathways.

## Supporting information

S1 Text Tables A–K. Table A in S1 Text. Sequences of primers and probes used for quantitative polymerase chain reaction. Table B in S1 Text. WASH characteristics of study population (N = 619). Table C in S1 Text. Environmental characteristics of study population (N = 830). Table D in S1 Text. Unadjusted STH prevalence by species, stratified by sex and age group. Table E in S1 Text. Mean eggs per gram (epg) of stool and incidence rate ratios (IRRs) by sex and age group for *T*. *trichiura* and *N*. *americanus* infections. Table F in S1 Text. Results of model building steps for *N*. *americanus* model. Table G in S1 Text. Results of model building steps for *A*. *ceylanicum* model. Table H in S1 Text. Results of model building steps for *T*. *trichiura* model. Table I in S1 Text. Results of model building steps for *Strongyloides* spp. model **Table J in S1 Text. Results of model building steps for hookworm (undifferentiated) model Table K in S1 Text. Results of model building steps for STH (undifferentiated) model**.
(DOCX)

S1 STROBE Checklist Checklist for cross-sectional studies. (DOCX)
