# Supplementary material for: Using quantitative PCR to identify opportunities to strengthen soil-transmitted helminth control in Solomon Islands: A cross-sectional epidemiological survey
Source: PLoS Negl Trop Dis. 2022 May 23;16(5):e0010350. doi: 10.1371/journal.pntd.0010350 (PMC9126411; doi:10.1371/journal.pntd.0010350)
Supplement: S1 Text — Table A in S1 Text. Sequences of primers and probes used for quantitative polymerase chain reaction. Table B in S1 Text. WASH characteristics of study population (N = 619). Table C in S1 Text. Environmental characteristics of study population (N = 830). Table D in S1 Text. Unadjusted STH prevalence by species, stratified by sex and age group. Table E in S1 Text. Mean eggs per gram (epg) of stool and incidence rate ratios (IRRs) by sex and age group for T. trichiura and N. americanus infections. Table F in S1 Text. Results of model building steps for N. americanus model. Table G in S1 Text. Results of model building steps for A. ceylanicum model. Table H in S1 Text. Results of model building steps for T. trichiura model. Table I in S1 Text. Results of model building steps for Strongyloides spp. model Table J in S1 Text. Results of model building steps for hookworm (undifferentiated) model Table K in S1 Text. Results of model building steps for STH (undifferentiated) model. (DOCX) [file pntd.0010350.s001.docx]

**Supporting Information (S1 Text)**

| **Supplementary Table A. Sequences of primers and probes used for quantitative polymerase chain reaction.** | | | |
| --- | --- | --- | --- |
| **Target** | **Oligonucleotide sequence (5’ to 3’)** | | **Reference** |
| ***Ascaris* spp. (ITS1)** | Forward | GTAATAGCAGTCGGCGGTTTCTT | Basuni et al., 2011 [1] |
|  | Reverse | GCCCAACATGCCACCTATTC | Basuni et al., 2011 [1] |
|  | Probe | /5HEX/TT GGC GGA C/ZEN/A ATT GCA TGC GAT /3IABkFQ/ | Modified from Basuni et al., 2011 [1] |
| ***Trichuris* spp. (18S)** | Forward | TTGAAACGACTTGCTCATCAACTT | Liu et al., 2013 [2] |
|  | Reverse | CTGATTCTCCGTTAACCGTTGTC | Liu et al., 2013 [2] |
|  | Probe | /5CY5/ CGATGGTAC/TAO/GCTACGTGCTTACCATGG- 3IAbRQSp | Modified from Liu et al., 2013 [2] |
| ***Strongyloides* spp. (18S)** | Forward | CCAAGTAAACGTAAGTCATTAGC | Zendejas-Heredia et al. 2021 [3] |
|  | Reverse | CGCCTCTGGATATTGCTCAGTTCC | Zendejas-Heredia et al. 2021 [3] |
|  | Probe | /56FAM/ACACACCGG/ZEN/CCGTCGCTGC/IBFQ | Modified from Verweij et al., 2009 [4] |
| **Equine Herpesvirus -4 (Glycoprotein gB)** | Forward | GATGACACTAGCGACTTCGA | Lambert et al., 2008 [5] |
|  | Reverse | CAGGGCAGAAACCATAGACA | Lambert et al., 2008 [5] |
|  | Probe | /ROX/TTTCGCGTGCCTCCTCCAG/3IAbRQSp/ | Modified from Lambert et al., 2008 [5] |
| ***Necator americanus* (ITS2)** | Forward | CTGTTTGTCGAACGGTACTTGC | Verweij et al., 2007 [6] |
|  | Reverse | ATAACAGCGTGCACATGTTGC | Verweij et al., 2007 [6] |
|  | Probe | 5/Cy5/CTG+TA+CTA+CG+CAT+TGTATAC–MGBNFQ | Modified from Verweij et al., 2007 [6] |
| ***Ancylostoma* spp.** | Forward | CGGGAAGGTTGGGAGTATC | Hii et al., 2018 [7] |
|  | Reverse | CGAACTTCGCACAGCAATC | Hii et al., 2018 [7] |
| ***A. ceylanicum*** | Probe | /56FAM/CCGTTC+CTGGGTGGC/3IABkFQ/ | Hii et al., 2018 [7] |
| ***A. duodenale*** | Probe | /5HEX/TCGTTAC+T+GGGTGACGG/3IABkFQ/ | Hii et al., 2018 [7] |
| **Human mt DNA** | Forward | CGACCTCGATGTTGGATCAG | Zendejas-Heredia et al. 2021 [3] |
|  | Reverse | GAACTCAGATCACGTAGGACTTT | Zendejas-Heredia et al. 2021 [3] |
|  | Probe | /ROX/CCCGATGGT/ZEN/GCAGCCGCTATTAAA/3IAbRQSp/ | Zendejas-Heredia et al. 2021 [3] |

“+”symbols found in the probe sequences indicate the presence of a Locked Nucleic Acid Base.

| **Supplementary Table B. WASH characteristics of study population (N=619).** | | |
| --- | --- | --- |
| **WASH questionnaire** | **Response options** | **n (%)** |
| *Where do you usually defecate?* | Bush or river | 27 (4.36) |
|  | Beach or ocean or mangrove | 487 (78.68) |
|  | Flush toilet or latrine | 105 (16.96) |
| *If the main place of defecation is latrine/ flush toilet, do you ever defecate on the ground?* | Yes | 65 (58.04) |
|  | No | 47 (41.96) |
| *Do you wash your hands with soap after going to the toilet?* | Never | 51 (8.24) |
|  | Sometimes | 503 (81.26) |
|  | Always | 57 (9.21) |
| *Do you wash your hands with soap before eating?* | Never | 43 (6.95) |
|  | Sometimes | 519 (83.84) |
|  | Always | 53 (8.56) |
| *Do you wear shoes when you are outside?* | Never | 199 (32.15) |
|  | Sometimes | 317 (51.21) |
|  | Always | 97 (15.67) |
| *Do you wear shoes when going to the toilet?* | Never | 211 (34.09) |
|  | Sometimes | 297 (47.98) |
|  | Always | 108 (17.45) |
| *Where does your drinking water come from?* | **Improved source**  (public tap/standpipe, protected spring, rainwater collection) | 420 (67.85) |
|  | **Unimproved source** (unprotected spring, unprotected well) | 198 (31.99) |
| *Does your house have a latrine/toilet?* | Yes | 126 (20.36) |
|  | No | 486 (78.51) |
| *If yes, can you flush the latrine/toilet with water?* | Yes | 106 (85.48) |
|  | No | 18 (14.52) |
| *If yes, does the latrine/toilet have a concrete floor?* | Yes | 107 (86.29) |
|  | No | 17 (13.71) |
| *Is there a place to wash your hands at your house?* | Yes | 554 (89.50) |
|  | No | 57 (9.21) |

| **Environmental variable** | **Mean (SD)** | **Range** |
| --- | --- | --- |
| Annual temperature (**°**C) | 26.88 (0.15) | 26.58 – 27.15 |
| Annual temperature range (**°**C) | 7.61 (0.10) | 7.40 – 7.80 |
| Max temperature in warmest month (**°**C) | 30.83 (0.21) | 30.40 – 31.2 |
| Min temperature in coldest month (**°**C) | 23.22 (0.14) | 23.0 – 23.4 |
| Temperature in wettest quarter (**°**C) | 27.17 (0.17) | 26.87 – 27.5 |
| Temperature in driest quarter (**°**C) | 26.98 (0.17) | 26.75 – 27.35 |
| Temperature in warmest quarter (**°**C) | 27.32 (0.19) | 26. 99 – 27.73 |
| Temperature in coldest quarter (**°**C) | 26.30 (0.14) | 26.03 – 26.48 |
| Annual precipitation (cm) | 341.77 (10.07) | 321.80 – 353.65 |
| Precipitation in wettest month (cm) | 38.57 (0.86) | 36.70 – 39.80 |
| Precipitation in driest month (cm) | 22.32 (1.73) | 18.1 – 23.99 |
| Precipitation in wettest quarter (cm) | 108.21 (1.43) | 105.28 – 112.00 |
| Precipitation in driest quarter (cm) | 72.52 (2.49) | 67.00 – 75.18 |
| Precipitation in warmest quarter (cm) | 87.41 (3.48) | 83.59 – 95.90 |
| Precipitation in coldest quarter (cm) | 83.93 (5.36) | 72.80 – 89.66 |
| Elevation (m) | 19.58 (7.92) | 6.73 – 34.34 |
| Slope (**°**) | 2.54 (1.09) | 1.16 – 4.82 |
| Vegetation (NDVI) | 0.64 (0.09) | 0.14 – 0.75 |
| Vegetation (EVI) | 0.42 (0.10) | 0.05 – 0.57 |
|  | **n (%)** |  |
| Evergreen broadleaf forest landcover | 540 (65.06) |  |
| Permanent wetlands landcover | 290 (34.94) |  |
| Clay soil texture | 423 (50.96) |  |
| Clay-loam soil texture | 407 (49.04) |  |
| Soil pH 5.20 – 5.30 | 346 (41.68) |  |
| Soil pH >5.30 – 5.50 | 484 (58.31) |  |

**Supplementary Table C. Environmental characteristics of study population (N=830).**

| **Supplementary Table D. Unadjusted STH prevalence by species, stratified by sex and age group.** | | | | | | | | | |
| --- | --- | --- | --- | --- | --- | --- | --- | --- | --- |
|  | **Study sample**  **n** | **Any STH**  **n (%)** | ***A. lumbricoides* n (%)** | ***T. trichiura***  **n (%)** | ***N. americanus***  **n (%)** | | ***A. ceylanicum***  **n (%)** | ***A. duodenale***  **n (%)** | ***Strongyloides* spp.**  **n (%)** |
| **Sex** | | | |  | |  | | | |
| **Male** | 352 | 228 (64.77) | 3 (0.85) | 72 (20.45) | 206 (58.52) | | 51 (14.49) | 0 | 25 (7.10) |
| **Female** | 478 | 291 (60.88) | 3 (0.63) | 100 (20.92) | 254 (53.14) | | 89 (18.62) | 0 | 24 (5.02) |
| **Age (years)** | | | |  | |  | | | |
| **1 – 5** | 212 | 89 (41.98) | 3 (1.42) | 30 (14.15) | 66 (31.13) | | 25 (11.79) | 0 | 4 (1.89) |
| **6 – 11** | 201 | 138 (68.66) | 1 (0.50) | 58 (28.86) | 124 (61.69) | | 37 (18.41) | 0 | 10 (4.98) |
| **12 – 17** | 89 | 70 (78.65) | 2 (2.25) | 32 (35.96) | 66 (74.16) | | 24 (26.97) | 0 | 14 (15.73) |
| **18 – 34** | 99 | 67 (67.68) | 0 | 21 (21.21) | 59 (59.60) | | 16 (16.16) | 0 | 7 (7.07) |
| **35 – 49** | 108 | 80 (74.07) | 0 | 21 (19.44) | 73 (67.59) | | 18 (16.67) | 0 | 8 (7.41) |
| **≥50** | 121 | 75 (61.98) | 0 | 10 (8.26) | 72 (59.50) | | 20 (16.53) | 0 | 6 (4.96) |
| **Total** | **830** | **519 (62.53)** | **6 (0.72)** | **172 (20.72)** | **460 (55.42)** | | **140 (16.87)** | **0** | **49 (5.90)** |

| **Supplementary Table E. Mean eggs per gram (epg) of stool and incidence rate ratios (IRRs) by sex and age group for *T. trichiura* and *N. americanus* infections.** | | | | |
| --- | --- | --- | --- | --- |
|  | ***T. trichiura*** | | ***N. americanus*** | |
|  | **Mean epg (SD)** | **IRR**  **(95% CI, *p*)** | **Mean epg**  **(SD)** | **IRR**  **(95% CI, *p*)** |
| **Sex** |  |  |  |  |
| **Male** | 319.81  (754.26) | 0.58  (0.31 – 1.10, *p*=0.093) | 913.05  (1610.30) | 1.03  (0.722 – 1.49, *p*=0.846) |
| **Female** | 591.95  (1500.46) | Reference group | 926.18  (1989.63) | Reference group |
| **Age (years)** |  |  |  |  |
| **1 – 5** | 569.13  (1537.89) | Reference group | 588.86 (1655.00) | Reference group |
| **6 – 11** | 815.43  (1679.39) | 1.94  (0.86 – 4.36, *p*=0.110) | 1107.96  (2125.12) | 2.62  (1.46 – 4.71, *p*=0.001) |
| **12 – 17** | 180.08  (313.36) | 0.72  (0.28 – 1.90, *p*=0.510) | 1654.62  (2488.30) | 4.04  (2.11 – 7.71, *p*<0.001) |
| **18 – 34** | 366.23  (980.61) | 0.62  (0.235 – 1.65, *p*=0.340) | 488.09  (788.31) | 1.52  (0.76 – 3.06, *p*=0.236) |
| **35 – 49** | 95.34  (171.50) | 0.42  (1.42 – 1.25, *p*=0.118) | 665.85  (1137.88) | 1.70  (0.90 – 3.21, *p*=0.101) |
| **≥50** | 239.73  (251.11) | 0.99  (0.23 – 4.22, *p*=0.991) | 839.53  (1697.02) | 2.55  (1.28 – 5.06, *p*=0.007) |

| **Supplementary Table F. Results of model building steps for *N. americanus* model.** | | | | | | | | | | | | | |
| --- | --- | --- | --- | --- | --- | --- | --- | --- | --- | --- | --- | --- | --- |
| Variables | Univariable regression  (Retain if p<0.20) | | Within domain multicollinearity  (Retain if VIF<5) | | Within domain analysis  (Retain if p<0.10) | | Between domain multicollinearity (Retain if VIF<5) | | Backward stepwise elimination  (p values of full model) | | Backward stepwise elimination  (p values of final model) | | Variables in final model |
|  | p value | Decision | VIF | Decision | p value | Decision | VIF | Decision | p value | Decision | p value | Decision |  |
| **Demographic variables** | | | | | | | | | | | | | |
| Sex | 0.043 | Retain | 1.01 | Retain | 0.008 | Retain | 1.07 | Retain | 0.014 | Retained | <0.001 | Retain | X |
| Age group | <0.001 | Retain | 1.01 | Retain | <0.001 | Retain | 1.04 | Retain | <0.001 | Retained | <0.001 | Retain | X |
| **Co-infection variables** | | | | | | | | | | | | | |
| *A. ceylanicum* | <0.001 | Retain | 1.02 | Retain | <0.001 | Retain | 1.06 | Retain | <0.001 | Retained | <0.001 | Retain | X |
| *T. trichiura* | <0.001 | Retain | 1.05 | Retain | <0.001 | Retain | 1.25 | Retain | 0.008 | Retained | 0.004 | Retain | X |
| *Strongyloides* spp. | 0.002 | Retain | 1.05 | Retain | 0.096 | Retain | 1.12 | Retain | 0.185 | **Removed 2nd** |  |  |  |
| **WASH variables** | | | | | | | | | | | | | |
| ***Individual sanitation*** | | | | | | | | | | | | | |
| Usual place of defecation | 0.002 | Retain | 1.18 | Retain | 0.476 | **Drop** |  |  |  |  |  |  |  |
| Practices open defecation | 0.186 | Retain | 1.12 | Retain | 0.284 | **Drop** |  |  |  |  |  |  |  |
| ***Individual hygiene*** | | | | | | | | | | | | | |
| Washes hand after defecation | 0.070 | Retain | 1.13 | Retain | 0.166 | **Drop** |  |  |  |  |  |  |  |
| Washes hand before eating | 0.433 | **Drop** |  |  |  |  |  |  |  |  |  |  |  |
| Wears shoes outside | 0.793 | **Drop** |  |  |  |  |  |  |  |  |  |  |  |
| Wears shoes when defecating | 0.964 | **Drop** |  |  |  |  |  |  |  |  |  |  |  |
| ***Household water*** | | | | | | | | | | | | | |
| Main drinking water source | 0.148 | Retain | 1.02 | Retain | 0.152 | **Drop** |  |  |  |  |  |  |  |
| ***Household sanitation*** | | | | | | | | | | | | | |
| HH has latrine | 0.007 | Retain | 1.19 | Retain | <0.001 | Retain | 1.01 | Retain | 0.008 | Retained | <0.001 | Retain | X |
| HH latrine can flush | 0.546 | **Drop** |  |  |  |  |  |  |  |  |  |  |  |
| HH latrine has slab | 0.946 | **Drop** |  |  |  |  |  |  |  |  |  |  |  |
| ***Household hygiene*** | | | | | | | | | | | | | |
| HH has handwashing station | 0.855 | **Drop** |  |  |  |  |  |  |  |  |  |  |  |

| **Supplementary Table F. Results of model building steps for *N. americanus* model (cont’d).** | | | | | | | | | | | | | |
| --- | --- | --- | --- | --- | --- | --- | --- | --- | --- | --- | --- | --- | --- |
| Variables | Univariable regression  (Retain if p<0.20) | | Within domain multicollinearity  (Retain if VIF <5) | | Within domain analysis  (Retain if p<0.10) | | Between domain multicollinearity (Retain if VIF <5) | | Backward stepwise elimination  (p values of full model) | | Backward stepwise elimination  (p values of final model) | | Vars in final model |
|  | p value | Decision | VIF | Decision | p value | Decision | VIF | Decision | p value | Decision | p value | Decision |  |
| **Temperature variables** | | | | | | | | | | | | | |
| Annual mean temp | 0.425 | **Drop** |  |  |  |  |  |  |  |  |  |  |  |
| Temp annual range | 0.964 | **Drop** |  |  |  |  |  |  |  |  |  |  |  |
| Max temp of warmest month | 0.282 | **Drop** |  |  |  |  |  |  |  |  |  |  |  |
| Min temp of coldest month | 0.089 | Retain | 1.00 | Retain | 0.119 | **Drop** |  |  |  |  |  |  |  |
| Mean temp of wettest quarter | 0.499 | **Drop** |  |  |  |  |  |  |  |  |  |  |  |
| Mean temp of driest quarter | 0.797 | **Drop** |  |  |  |  |  |  |  |  |  |  |  |
| Mean temp of warmest quarter | 0.504 | **Drop** |  |  |  |  |  |  |  |  |  |  |  |
| Mean temp of coldest quarter | 0.334 | **Drop** |  |  |  |  |  |  |  |  |  |  |  |
| **Elevation variables** | | | | | | | | | | | | | |
| Elevation | 0.041 | Retain | 1.00 | Retain | 0.086 | Retain | 1.42 | Retain | 0.920 | **Remove 1st** |  |  |  |
| Slope | 0.579 | **Drop** |  |  |  |  |  |  |  |  |  |  |  |
| **Precipitation variables** | | | | | | | | | | | | | |
| Annual precipitation | 0.499 | **Drop** |  |  |  |  |  |  |  |  |  |  |  |
| Prec of wettest month | 0.100 | Retain | 1.05 | Retain | 0.148 | **Drop** |  |  |  |  |  |  |  |
| Prec of driest month | 0.753 | **Drop** |  |  |  |  |  |  |  |  |  |  |  |
| Prec of wettest quarter | 0.451 | **Drop** |  |  |  |  |  |  |  |  |  |  |  |
| Prec of driest quarter | 0.969 | **Drop** |  |  |  |  |  |  |  |  |  |  |  |
| Prec of warmest quarter | 0.069 | Retain | 1.05 | Retain | 0.126 | **Drop** |  |  |  |  |  |  |  |
| Prec of coldest quarter | 0.323 | **Drop** |  |  |  |  |  |  |  |  |  |  |  |
| **Soil composition** | | | | | | | | | | | | | |
| Soil texture | 0.417 | **Drop** |  |  |  |  |  |  |  |  |  |  |  |
| Soil pH class | <0.001 | Retain | 1.00 | Retain | 0.001 | Retain | 1.24 | Retain | 0.026 | Retain | 0.010 | Retain | X |
| **Landcover/ vegetation** | | | | | | | | | | | | | |
| Landcover type | 0.402 | **Drop** |  |  |  |  |  |  |  |  |  |  |  |
| NDVI | 0.021 | Retain | 3.01 | Retain | 0.445 | **Drop** |  |  |  |  |  |  |  |
| EVI | 0.041 | Retain | 3.01 | Retain | 0.798 | **Drop** |  |  |  |  |  |  |  |

| **Supplementary Table G. Results of model building steps for *A. ceylanicum* model.** | | | | | | | | | | | | | |
| --- | --- | --- | --- | --- | --- | --- | --- | --- | --- | --- | --- | --- | --- |
| Variables | Univariable regression  (Retain if p<0.20) | | Within domain multicollinearity  (Retain if VIF<5) | | Within domain analysis  (Retain if p<0.10) | | Between domain multicollinearity (Retain if VIF<5) | | Backward stepwise elimination  (p values of full model) | | Backward stepwise elimination  (p values of final model) | | Variables in final model |
|  | p value | Decision | VIF | Decision | p value | Decision | VIF | Decision | p value | Decision | p value | Decision |  |
| **Demographic variables** | | | | | | | | | | | | | |
| Sex | 0.256 | Retain anyway | 1.01 | Retain | 0.212 | Retain anyway | 1.08 | Retain | 0.017 | Retain | 0.013 | Retain | X |
| Age group | 0.094 | Retain | 1.01 | Retain | 0.084 | Retain | 1.19 | Retain | 0.528 | Retain anyway | 0.533 | Retain anyway | X |
| **Co-infection variables** | | | | | | | | | | | | | |
| *N. americanus* | <0.001 | Retain | 1.10 | Retain | <0.001 | Retain | 1.19 | Retain | <0.001 | Retain | <0.001 | Retain | X |
| *T. trichiura* | 0.002 | Retain | 1.11 | Retain | 0.120 | **Drop** |  |  |  |  |  |  |  |
| *Strongyloides* spp. | 0.008 | Retain | 1.06 | Retain | 0.090 | Retain | 1.07 | Retain | 0.013 | Retain | 0.006 | Retain | X |
| **WASH variables** | | | | | | | | | | | | | |
| ***Individual sanitation*** | | | | | | | | | | | | | |
| Usual place of defecation | 0.236 | **Drop** |  |  |  |  |  |  |  |  |  |  |  |
| Practices open defecation | 0.254 | **Drop** |  |  |  |  |  |  |  |  |  |  |  |
| ***Individual hygiene*** | | | | | | | | | | | | | |
| Washes hand after defecation | 0.065 | Retain | 1.05 | Retain | 0.024 | Retain | 1.09 | Retain | 0.027 | **Removed 3rd** |  |  |  |
| Washes hand before eating | 0.485 | Drop |  |  |  |  |  |  |  |  |  |  |  |
| Wears shoes outside | 0.099 | Retain | 5.49 | Retain  (lower AIC) | 0.075 | Retain | 1.20 | Retain | 0.098 | **Removed 2nd** |  |  |  |
| Wears shoes when defecating | 0.155 | Retain | 5.47 | **Drop** |  |  |  |  |  |  |  |  |  |
| **Household water** | | | | | | | | | | | | | |
| Main drinking water source | 0.033 | Retain | 1.01 | Retain | 0.035 | Retain | 1.11 | Retain | <0.001 | Retain | 0.003 | Retain | X |
| **Household sanitation** | | | | | | | | | | | | | |
| HH has latrine | 0.222 | **Drop** |  |  |  |  |  |  |  |  |  |  |  |
| HH latrine can flush | Low n | **Drop** |  |  |  |  |  |  |  |  |  |  |  |
| HH latrine has slab | Low n | **Drop** |  |  |  |  |  |  |  |  |  |  |  |
| **Household hygiene** | | | | | | | | | | | | | |
| HH has handwashing station | 0.250 | **Drop** |  |  |  |  |  |  |  |  |  |  |  |

| **Supplementary Table G. Results of model building steps for *A. ceylanicum* model (cont’d).** | | | | | | | | | | | | | |
| --- | --- | --- | --- | --- | --- | --- | --- | --- | --- | --- | --- | --- | --- |
| Variables | Univariable regression  (Retain if p<0.20) | | Within domain multicollinearity  (Retain if VIF<5) | | Within domain analysis  (Retain if p<0.10) | | Between domain multicollinearity (Retain if VIF<5) | | Backward stepwise elimination  (p values of full model) | | Backward stepwise elimination  (p values of final model) | | Variables in final model |
|  | p value | Decision | VIF | Decision | p value | Decision | VIF | Decision | p value | Decision | p value | Decision |  |
| **Temperature variables** | | | | | | | | | | | | | |
| Annual mean temp | 0.087 | Retain | >100 | Retain^1^ | 0.778 | **Drop** |  |  |  |  |  |  |  |
| Temp annual range | 0.038 | Retain | >100 | Retain^1^ | 0.219 | **Drop** |  |  |  |  |  |  |  |
| Max temp of warmest month | 0.051 | Retain | >100 | **Drop^1^** |  |  |  |  |  |  |  |  |  |
| Min temp of coldest month | 0.146 | Retain | >100 | **Drop^1^** |  |  |  |  |  |  |  |  |  |
| Mean temp of wettest quarter | 0.159 | Retain | >100 | **Drop^1^** |  |  |  |  |  |  |  |  |  |
| Mean temp of driest quarter | 0.696 | Drop |  |  |  |  |  |  |  |  |  |  |  |
| Mean temp of warmest quarter | 0.045 | Retain | >100 | **Drop^1^** |  |  |  |  |  |  |  |  |  |
| Mean temp of coldest quarter | 0.182 | Retain | >100 | **Drop^1^** |  |  |  |  |  |  |  |  |  |
| **Elevation variables** | | | | | | | | | | | | | |
| Elevation | 0.526 | **Drop** |  |  |  |  |  |  |  |  |  |  |  |
| Slope | 0.701 | **Drop** |  |  |  |  |  |  |  |  |  |  |  |
| **Precipitation variables** | | | | | | | | | | | | | |
| Annual precipitation (prec) | 0.305 | **Drop** |  |  |  |  |  |  |  |  |  |  |  |
| Prec of wettest month | 0.601 | **Drop** |  |  |  |  |  |  |  |  |  |  |  |
| Prec of driest month | 0.250 | **Drop** |  |  |  |  |  |  |  |  |  |  |  |
| Prec of wettest quarter | 0.719 | **Drop** |  |  |  |  |  |  |  |  |  |  |  |
| Prec of driest quarter | 0.209 | **Drop** |  |  |  |  |  |  |  |  |  |  |  |
| Prec of warmest quarter | 0.499 | **Drop** |  |  |  |  |  |  |  |  |  |  |  |
| Prec of coldest quarter | 0.342 | **Drop** |  |  |  |  |  |  |  |  |  |  |  |
| **Soil composition** | | | | | | | | | | | | | |
| Soil texture | 0.559 | **Drop** |  |  |  |  |  |  |  |  |  |  |  |
| Soil pH class | 0.009 | Retain | 1.00 | Retain | 0.012 | Retain | 1.62 | Retain | 0.348 | **Removed 1st** |  |  |  |
| **Landcover/vegetation** | | | | | | | | | | | | | |
| Landcover type | 0.010 | Retain | 1.27 | Retain | 0.050 | Retain | 1.84 | Retain | 0.236 | **Removed 4th** |  |  |  |
| NDVI | 0.123 | Retain | 3.22 | Retain | 0.900 | **Drop** |  |  |  |  |  |  |  |
| EVI | 0.123 | Retain | 3.02 | Retain | 0.719 | **Drop** |  |  |  |  |  |  |  |
| **^1^**Variables were iteratively removed from the model, in descending order of VIF, then re-tested for multicollinearity until all retained variables had VIF<5. | | | | | | | | | | | | | |

| **Supplementary Table H. Results of model building steps for *T. trichiura* model.** | | | | | | | | | | | | | |
| --- | --- | --- | --- | --- | --- | --- | --- | --- | --- | --- | --- | --- | --- |
| Variables | Univariable regression  (Retain if p<0.20) | | Within domain multicollinearity  (Retain if VIF<5) | | Within domain analysis  (Retain if p<0.10) | | Between domain multicollinearity (Retain if VIF<5) | | Backward stepwise elimination  (p values of full model) | | Backward stepwise elimination  (p values of final model) | | Variables in final model |
|  | p value | Decision | VIF | Decision | p value | Decision | VIF | Decision | p value | Decision | p value | Decision |  |
| **Demographic variables** | | | | | | | | | | | | | |
| Sex | 0.552 | Retain anyway | 1.01 | Retain | 0.643 | Retain anyway | 1.08 | Retain | 0.579 | Retain anyway | 0.688 | Retain anyway | X |
| Age group | <0.001 | Retain | 1.01 | Retain | <0.001 | Retain | 1.18 | Retain | 0.027 | Retain | 0.028 | Retain | X |
| **Co-infection variables** | | | | | | | | | | | | | |
| *A. ceylanicum* | 0.002 | Retain | 1.06 | Retain | 0.142 | **Drop** |  |  |  |  |  |  |  |
| *N. americanus* | <0.001 | Retain | 1.07 | Retain | <0.001 | Retain | 1.09 | Retain | 0.002 | Retain | <0.001 | Retain | X |
| *Strongyloides* spp. | 0.016 | Retain | 1.04 | Retain | 0.144 | **Drop** |  |  |  |  |  |  |  |
| **WASH variables** | | | | | | | | | | | | | |
| ***Individual sanitation*** | | | | | | | | | | | | | |
| Usual place of defecation | 0.248 | **Drop** |  |  |  |  |  |  |  |  |  |  |  |
| Practices open defecation | 0.688 | **Drop** |  |  |  |  |  |  |  |  |  |  |  |
| ***Individual hygiene*** | | | | | | | | | | | | | |
| Washes hand after defecation | 0.119 | Retain | 1.06 | Retain | 0.233 | **Drop** |  |  |  |  |  |  |  |
| Washes hand before eating | 0.532 | Drop |  |  |  |  |  |  |  |  |  |  |  |
| Wears shoes outside | 0.015 | Retain | 5.50 | Retain (lower AIC) | 0.053 | Retain | 1.07 | Retain | 0.048 | Retain | 0.035 | Retain | X |
| Wears shoes when defecating | 0.039 | Retain | 5.50 | **Drop** |  |  |  |  |  |  |  |  |  |
| ***Household water*** | | | | | | | | | | | | | |
| Main drinking water source | 0.985 | **Drop** |  |  |  |  |  |  |  |  |  |  |  |
| ***Household sanitation*** | | | | | | | | | | | | | |
| HH has latrine | 0.104 | Retain | 1.01 | Retain | 0.087 | Retain | 1.01 | Retain | 0.229 | **Remove 3rd** |  |  |  |
| HH latrine can flush | Low n | **Drop** |  |  |  |  |  |  |  |  |  |  |  |
| HH latrine has slab | 0.688 | **Drop** |  |  |  |  |  |  |  |  |  |  |  |
| ***Household hygiene*** | | | | | | | | | | | | | |
| HH has handwashing station | 0.716 | **Drop** |  |  |  |  |  |  |  |  |  |  |  |

| **Supplementary Table H. Results of model building steps for *T. trichiura* model (cont’d).** | | | | | | | | | | | | | |
| --- | --- | --- | --- | --- | --- | --- | --- | --- | --- | --- | --- | --- | --- |
| Variables | Univariable regression  (Retain if p<0.20) | | Within domain multicollinearity  (Retain if VIF<5) | | Within domain analysis  (Retain if p<0.10) | | Between domain multicollinearity (Retain if VIF<5) | | Backward stepwise elimination  (p values of full model) | | Backward stepwise elimination  (p values of final model) | | Variables in final model |
|  | p value | Decision | VIF | Decision | p value | Decision | VIF | Decision | p value | Decision | p value | Decision |  |
| **Temperature variables** | | | | | | | | | | | | | |
| Annual mean temp | 0.219 | **Drop** |  |  |  |  |  |  |  |  |  |  |  |
| Temp annual range | 0.847 | **Drop** |  |  |  |  |  |  |  |  |  |  |  |
| Max temp of warmest month | 0.303 | **Drop** |  |  |  |  |  |  |  |  |  |  |  |
| Min temp of coldest month | 0.165 | Retain | 10.05 | **Drop** |  |  |  |  |  |  |  |  |  |
| Mean temp of wettest quarter | 0.396 | **Drop** |  |  |  |  |  |  |  |  |  |  |  |
| Mean temp of driest quarter | 0.813 | **Drop** |  |  |  |  |  |  |  |  |  |  |  |
| Mean temp of warmest quarter | 0.266 | **Drop** |  |  |  |  |  |  |  |  |  |  |  |
| Mean temp of coldest quarter | 0.082 | Retain | 10.05 | Retain (lower AIC) | **0.092** | **Retain** | **1.22** | **Retain** | **0.587** | **Remove 2nd** |  |  |  |
| **Elevation variables** | | | | | | | | | | | | | |
| Elevation | 0.012 | Retain | 1.00 | Retain | 0.011 | Retain | 2.35 | Retain | 0.714 | **Remove 1st** |  |  |  |
| Slope | 0.249 | Drop |  |  |  |  |  |  |  |  |  |  |  |
| **Precipitation** | | | | | | | | | | | | | |
| Annual precipitation (prec) | 0.005 | Retain | 22.78 | Retain (lowest AIC) | 0.004 | Retain | 1.21 | Retain | 0.169 | Retain | 0.008 | Retain | X |
| Prec of wettest month | 0.988 | Drop |  |  |  |  |  |  |  |  |  |  |  |
| Prec of driest month | 0.015 | Retain | 13.80 | **Drop** |  |  |  |  |  |  |  |  |  |
| Prec of wettest quarter | 0.588 | Drop |  |  |  |  |  |  |  |  |  |  |  |
| Prec of driest quarter | 0.023 | Retain | 18.03 | **Drop** |  |  |  |  |  |  |  |  |  |
| Prec of warmest quarter | 0.974 | Drop |  |  |  |  |  |  |  |  |  |  |  |
| Prec of coldest quarter | 0.015 | Retain | 15.60 | **Drop** |  |  |  |  |  |  |  |  |  |
| **Soil composition** | | | | | | | | | | | | | |
| Soil texture | 0.931 | **Drop** |  |  |  |  |  |  |  |  |  |  |  |
| Soil pH class | 0.141 | Retain | NA | Retain | 0.152 | **Drop** |  |  |  |  |  |  |  |
| **Landcover/vegetation** | | | | | | | | | | | | | |
| Landcover type | 0.124 | Retain | NA | Retain | 0.121 | **Drop** |  |  |  |  |  |  |  |
| NDVI | 0.974 | **Drop** |  |  |  |  |  |  |  |  |  |  |  |
| EVI | 0.365 | **Drop** |  |  |  |  |  |  |  |  |  |  |  |

| **Supplementary Table I. Results of model building steps for *Strongyloides* spp. model.** | | | | | | | | | | | | | |
| --- | --- | --- | --- | --- | --- | --- | --- | --- | --- | --- | --- | --- | --- |
| Variables | Univariable regression  (Retain if p<0.20) | | Within domain multicollinearity  (Retain if VIF<5) | | Within domain analysis  (Retain if p<0.10) | | Between domain multicollinearity (Retain if VIF<5) | | Backward stepwise elimination  (p values of full model) | | Backward stepwise elimination  (p values of final model) | | Variables in final model |
|  | p value | Decision | VIF | Decision | p value | Decision | VIF | Decision | p value | Decision | p value | Decision |  |
| **Demographic variables** | | | | | | | | | | | | | |
| Sex | 0.150 | Retain | 1.01 | Retain | 0.098 | Retain | 1.07 | Retain | 0.113 | Retain anyway | 0.149 | Retain | X |
| Age group | 0.032 | Retain | 1.01 | Retain | 0.026 | Retain | 1.09 | Retain | 0.077 | Retain anyway | 0.049 | Retain | X |
| **Co-infection variables** | | | | | | | | | | | | | |
| *N. Americanus* | 0.001 | Retain | 1.12 | Retain | 0.020 | Retain | 1.20 | Retain | 0.074 | Retain | 0.013 | Retain | X |
| *A. ceylanicum* | 0.015 | Retain | 1.05 | Retain | 0.080 | Retain | 1.11 | Retain | 0.031 | **Removed 4^th^** |  |  |  |
| *T. trichiura* | 0.004 | Retain | 1.08 | Retain | 0.022 | Retain | 1.25 | Retain | 0.045 | Retain | 0.026 | Retain | X |
| **WASH variables** | | | | | | | | | | | | | |
| ***Individual sanitation*** | | | | | | | | | | | | | |
| Usual place of defecation | 0.379 | **Drop** |  |  |  |  |  |  |  |  |  |  |  |
| Practices open defecation | 0.388 | **Drop** |  |  |  |  |  |  |  |  |  |  |  |
| ***Individual hygiene*** | | | | | | | | | | | | | |
| Washes hand after defecation | 0.901 | **Drop** |  |  |  |  |  |  |  |  |  |  |  |
| Washes hand before eating | 0.427 | **Drop** |  |  |  |  |  |  |  |  |  |  |  |
| Wears shoes outside | 0.507 | **Drop** |  |  |  |  |  |  |  |  |  |  |  |
| Wears shoes when defecating | 0.550 | **Drop** |  |  |  |  |  |  |  |  |  |  |  |
| ***Household water*** | | | | | | | | | | | | | |
| Main drinking water source | 0.089 | Retain | 1.00 | Retain | 0.068 | Retain | 1.17 | Retain | 0.163 | **Removed 3^rd^** |  |  |  |
| ***Household sanitation*** | | | | | | | | | | | | | |
| HH has latrine | 0.273 | **Drop** |  |  |  |  |  |  |  |  |  |  |  |
| HH latrine can flush | Low n | **Drop** |  |  |  |  |  |  |  |  |  |  |  |
| HH latrine has slab | Low n | **Drop** |  |  |  |  |  |  |  |  |  |  |  |
| ***Household hygiene*** | | | | | | | | | | | | | |
| HH has handwashing station | 0.993 | **Drop** |  |  |  |  |  |  |  |  |  |  |  |

| **Supplementary Table I. Results of model building steps for *Strongyloides* spp. model (cont’d).** | | | | | | | | | | | | | |
| --- | --- | --- | --- | --- | --- | --- | --- | --- | --- | --- | --- | --- | --- |
| Variables | Univariable regression  (Retain if p<0.20) | | Within domain multicollinearity  (Retain if VIF<5) | | Within domain analysis  (Retain if p<0.10) | | Between domain multicollinearity (Retain if VIF<5) | | Backward stepwise elimination  (p values of full model) | | Backward stepwise elimination  (p values of final model) | | Variables in final model |
|  | p value | Decision | VIF | Decision | p value | Decision | VIF | Decision | p value | Decision | p value | Decision |  |
| **Temperature variables** | | | | | | | | | | | | | |
| Annual mean temp | 0.061 | Retain | >100 | **Drop** |  |  |  |  |  |  |  |  |  |
| Temp annual range | 0.499 | **Drop** |  |  |  |  |  |  |  |  |  |  |  |
| Max temp of warmest month | 0.051 | Retain | 17.99 | **Drop** |  |  |  |  |  |  |  |  |  |
| Min temp of coldest month | 0.010 | Retain | 12.71 | Retain (lowest AIC) | 0.012 | Retain | 1.45 | Retain | 0.956 | **Removed 1st** |  |  |  |
| Mean temp of wettest quarter | 0.145 | **Drop** |  |  |  |  |  |  |  |  |  |  |  |
| Mean temp of driest quarter | 0.465 | **Drop** |  |  |  |  |  |  |  |  |  |  |  |
| Mean temp of warmest quarter | 0.070 | Retain | >100 | **Drop** |  |  |  |  |  |  |  |  |  |
| Mean temp of coldest quarter | 0.028 | Retain | 57.52 | **Drop** |  |  |  |  |  |  |  |  |  |
| **Elevation variables** | | | | | | | | | | | | | |
| Elevation | 0.240 | **Drop** |  |  |  |  |  |  |  |  |  |  |  |
| Slope | 0.250 | **Drop** |  |  |  |  |  |  |  |  |  |  |  |
| **Precipitation variables** | | | | | | | | | | | | | |
| Annual precipitation (prec) | 0.058 | Retain | 20.34 | **Drop** |  |  |  |  |  |  |  |  |  |
| Prec of wettest month | 0.260 | **Drop** |  |  |  |  |  |  |  |  |  |  |  |
| Prec of driest month | 0.219 | **Drop** |  |  |  |  |  |  |  |  |  |  |  |
| Prec of wettest quarter | 0.219 | **Drop** |  |  |  |  |  |  |  |  |  |  |  |
| Prec of driest quarter | 0.094 | Retain | 8.89 | **Drop** |  |  |  |  |  |  |  |  |  |
| Prec of warmest quarter | 0.368 | Drop |  |  |  |  |  |  |  |  |  |  |  |
| Prec of coldest quarter | 0.028 | Retain | 14.57 | Retain  (lowest AIC) | 0.027 | Retain | 1.13 | Retain | 0.370 | **Removed 2^nd^** |  |  |  |
| **Soil composition** | | | | | | | | | | | | | |
| Soil texture | 0.731 | **Drop** |  |  |  |  |  |  |  |  |  |  |  |
| Soil pH | 0.001 | Retain | 1.00 | Retain | 0.001 | Retain | 1.83 | Retain | 0.061 | Retain | 0.004 | Retain | X |
| **Landcover/ vegetation** | | | | | | | | | | | | | |
| Landcover type | 0.131 | Retain | 1.27 | Retain | 0.945 | **Drop** |  |  |  |  |  |  |  |
| NDVI | <0.001 | Retain | 3.22 | Retain | 0.235 | **Drop** |  |  |  |  |  |  |  |
| EVI | <0.001 | Retain | 3.02 | Retain | 0.294 | **Drop** |  |  |  |  |  |  |  |

| **Supplementary Table J. Results of model building steps for hookworm (undifferentiated) model.** | | | | | | | | | | | | | |
| --- | --- | --- | --- | --- | --- | --- | --- | --- | --- | --- | --- | --- | --- |
| Variables | Univariable regression  (Retain if p<0.20) | | Within domain multicollinearity  (Retain if VIF<5) | | Within domain analysis  (Retain if p<0.10) | | Between domain multicollinearity (Retain if VIF<5) | | Backward stepwise elimination  (p values of full model) | | Backward stepwise elimination  (p values of final model) | | Variables in final model |
|  | p value | Decision | VIF | Decision | p value | Decision | VIF | Decision | p value | Decision | p value | Decision |  |
| **Demographic variables** | | | | | | | | | | | | | |
| Sex | 0.043 | Retain | 1.01 | Retain | <0.001 | Retain | 1.06 | Retain | 0.101 | Retain anyway | 0.100 | Retain anyway | X |
| Age group | <0.001 | Retain | 1.01 | Retain | <0.001 | Retain | 1.03 | Retain | <0.001 | Retain | <0.001 | Retain | X |
| **Co-infection variables** | | | | | | | | | | | | | |
| *T. trichiura* | <0.001 | Retain | 1.00 | Retain | <0.001 | Retain | 1.20 | Retain | 0.001 | Retain | 0.001 | Retain | X |
| **WASH variables** | | | | | | | | | | | | | |
| ***Individual sanitation*** | | | | | | | | | | | | | |
| Usual place of defecation | 0.006 | Retain | 1.17 | Retain | 0.673 | **Drop** |  |  |  |  |  |  |  |
| Practices open defecation | 0.093 | Retain | 1.13 | Retain | 0.168 | **Drop** |  |  |  |  |  |  |  |
| ***Individual hygiene*** | | | | | | | | | | | | | |
| Washes hand after defecation | 0.038 | Retain | 1.12 | Retain | 0.120 | **Drop** |  |  |  |  |  |  |  |
| Washes hand before eating | 0.336 | **Drop** |  |  |  |  |  |  |  |  |  |  |  |
| Wears shoes outside | 0.471 | **Drop** |  |  |  |  |  |  |  |  |  |  |  |
| Wears shoes when defecating | 0.693 | **Drop** |  |  |  |  |  |  |  |  |  |  |  |
| ***Household water*** | | | | | | | | | | | | | |
| Main drinking water source | 0.266 | **Drop** |  |  |  |  |  |  |  |  |  |  |  |
| ***Household sanitation*** | | | | | | | | | | | | | |
| HH has latrine | 0.009 | Retain | 1.15 | Retain | 0.001 | Retain | 1.01 | Retain | 0.001 | Retain | 0.002 | Retain | X |
| HH latrine can flush | 0.372 | **Drop** |  |  |  |  |  |  |  |  |  |  |  |
| HH latrine has slab | 0.915 | **Drop** |  |  |  |  |  |  |  |  |  |  |  |
| ***Household hygiene*** | | | | | | | | | | | | | |
| HH has handwashing station | 0.462 | **Drop** |  |  |  |  |  |  |  |  |  |  |  |

| **Supplementary Table J. Results of model building steps for hookworm (undifferentiated) model (cont’d).** | | | | | | | | | | | | | |
| --- | --- | --- | --- | --- | --- | --- | --- | --- | --- | --- | --- | --- | --- |
| Variables | Univariable regression  (Retain if p<0.20) | | Within domain multicollinearity  (Retain if VIF<5) | | Within domain analysis  (Retain if p<0.10) | | Between domain multicollinearity (Retain if VIF<5) | | Backward stepwise elimination  (p values of full model) | | Backward stepwise elimination  (p values of final model) | | Variables in final model |
|  | p value | Decision | VIF | Decision | p value | Decision | VIF | Decision | p value | Decision | p value | Decision |  |
| **Temperature variables** | | | | | | | | | | | | | |
| Annual mean temp | 0.321 | **Drop** |  |  |  |  |  |  |  |  |  |  |  |
| Temp annual range | 0.909 | **Drop** |  |  |  |  |  |  |  |  |  |  |  |
| Max temp of warmest month | 0.199 | Retain | 6.90 | **Drop** |  |  |  |  |  |  |  |  |  |
| Min temp of coldest month | 0.056 | Retain | 6.90 | Retain (lower AIC) | 0.075 | Retain | 1.96 | Retain | 0.808 | **Removed 2nd** |  |  |  |
| Mean temp of wettest quarter | 0.390 | **Drop** |  |  |  |  |  |  |  |  |  |  |  |
| Mean temp of driest quarter | 0.679 | **Drop** |  |  |  |  |  |  |  |  |  |  |  |
| Mean temp of warmest quarter | 0.369 | **Drop** |  |  |  |  |  |  |  |  |  |  |  |
| Mean temp of coldest quarter | 0.265 | **Drop** |  |  |  |  |  |  |  |  |  |  |  |
| **Elevation variables** | | | | | | | | | | | | | |
| Elevation | 0.042 | Retain | 1.00 | Retain | 0.083 | Retain | 1.44 | Retain | 0.929 | **Removed 1st** |  |  |  |
| Slope | 0.620 | **Drop** |  |  |  |  |  |  |  |  |  |  |  |
| **Precipitation variables** | | | | | | | | | | | | | |
| Annual precipitation (prec) | 0.496 | **Drop** |  |  |  |  |  |  |  |  |  |  |  |
| Prec of wettest month | 0.107 | Retain | 1.05 | Retain | 0.156 | **Drop** |  |  |  |  |  |  |  |
| Prec of driest month | 0.791 | **Drop** |  |  |  |  |  |  |  |  |  |  |  |
| Prec of wettest quarter | 0.399 | **Drop** |  |  |  |  |  |  |  |  |  |  |  |
| Prec of driest quarter | 0.925 | **Drop** |  |  |  |  |  |  |  |  |  |  |  |
| Prec of warmest quarter | 0.094 | Retain | 1.05 | Retain | 0.157 | **Drop** |  |  |  |  |  |  |  |
| Prec of coldest quarter | 0.302 | **Drop** |  |  |  |  |  |  |  |  |  |  |  |
| **Soil composition** | | | | | | | | | | | | | |
| Soil texture | 0.316 | **Drop** |  |  |  |  |  |  |  |  |  |  |  |
| Soil pH | <0.001 | Retain | 1.00 | Retain | 0.001 | Retain | 1.74 | Retain | 0.033 | Retain | 0.011 | Retain | X |
| **Landcover/ vegetation** | | | | | | | | | | | | | |
| Landcover type | 0.298 | **Drop** |  |  |  |  |  |  |  |  |  |  |  |
| NDVI | 0.013 | Retain | 3.01 | Retain | 0.270 | **Drop** |  |  |  |  |  |  |  |
| EVI | 0.039 | Retain | 3.01 | Retain | 0.998 | **Drop** |  |  |  |  |  |  |  |

| **Supplementary Table K. Results of model building steps for STH (undifferentiated) model.** | | | | | | | | | | | | | |
| --- | --- | --- | --- | --- | --- | --- | --- | --- | --- | --- | --- | --- | --- |
| Variables | Univariable regression  (Retain if p<0.20) | | Within domain multicollinearity  (Retain if VIF<5) | | Within domain analysis  (Retain if p<0.10) | | Between domain multicollinearity (Retain if VIF<5) | | Backward stepwise elimination  (p values of full model) | | Backward stepwise elimination  (p values of final model) | | Variables in final model |
|  | p value | Decision | VIF | Decision | p value | Decision | VIF | Decision | p value | Decision | p value | Decision |  |
| **Demographic variables** | | | | | | | | | | | | | |
| Sex | 0.090 | Retain | 1.01 | Retain | 0.033 | Retain | 1.06 | Retain | 0.086 | Retain anyway | 0.089 | Retain anyway | X |
| Age group | <0.001 | Retain | 1.01 | Retain | <0.001 | Retain | 1.04 | Retain | <0.001 | Retain | <0.001 | Retain | X |
| **WASH variables** | | | | | | | | | | | | | |
| ***Individual sanitation*** | | | | | | | | | | | | | |
| Usual place of defecation | 0.004 | Retain | 1.17 | Retain | 0.877 | **Drop** |  |  |  |  |  |  |  |
| Practices open defecation | 0.124 | Retain | 1.14 | Retain | 0.166 | **Drop** |  |  |  |  |  |  |  |
| ***Individual hygiene*** | | | | | | | | | | | | | |
| Washes hand after defecation | 0.032 | Retain | 1.79 | Retain | 0.134 | **Drop** |  |  |  |  |  |  |  |
| Washes hand before eating | 0.084 | Retain | 1.75 | Retain | 0.131 | **Drop** |  |  |  |  |  |  |  |
| Wears shoes outside | 0.273 | **Drop** |  |  |  |  |  |  |  |  |  |  |  |
| Wears shoes when defecating | 0.581 | **Drop** |  |  |  |  |  |  |  |  |  |  |  |
| ***Household water*** | | | | | | | | | | | | | |
| Main drinking water source | 0.105 | Retain | 1.04 | Retain | 0.115 | **Drop** |  |  |  |  |  |  |  |
| ***Household sanitation*** | | | | | | | | | | | | | |
| HH has latrine | 0.008 | Retain | 1.20 | Retain | 0.001 | Retain | 1.02 | Retain | 0.001 | Retain | 0.001 | Retain | X |
| HH latrine can flush | 0.234 | **Drop** |  |  |  |  |  |  |  |  |  |  |  |
| HH latrine has slab | 0.804 | **Drop** |  |  |  |  |  |  |  |  |  |  |  |
| ***Household hygiene*** | | | | | | | | | | | | | |
| HH has handwashing station | 0.200 | **Drop** |  |  |  |  |  |  |  |  |  |  |  |

| **Supplementary Table K. Results of model building steps for STH (undifferentiated) model (cont’d).** | | | | | | | | | | | | | |
| --- | --- | --- | --- | --- | --- | --- | --- | --- | --- | --- | --- | --- | --- |
| Variables | Univariable regression  (Retain if p<0.20) | | Within domain multicollinearity  (Retain if VIF<5) | | Within domain analysis  (Retain if p<0.10) | | Between domain multicollinearity (Retain if VIF<5) | | Backward stepwise elimination  (p values of full model) | | Backward stepwise elimination  (p values of final model) | | Variables in final model |
|  | p value | Decision | VIF | Decision | p value | Decision | VIF | Decision | p value | Decision | p value | Decision |  |
| **Temperature variables** | | | | | | | | | | | | | |
| Annual mean temp | 0.199 | Retain | 32.75 | **Drop** |  |  |  |  |  |  |  |  |  |
| Temp annual range | 0.716 | **Drop** |  |  |  |  |  |  |  |  |  |  |  |
| Max temp of warmest month | 0.118 | Retain | 17.00 | **Drop** |  |  |  |  |  |  |  |  |  |
| Min temp of coldest month | 0.031 | Retain | 12.68 | Retain (lowest AIC) | 0.039 | Retain | 3.23 | Retain | 0.966 | **Removed 1st** |  |  |  |
| Mean temp of wettest quarter | 0.279 | **Drop** |  |  |  |  |  |  |  |  |  |  |  |
| Mean temp of driest quarter | 0.691 | **Drop** |  |  |  |  |  |  |  |  |  |  |  |
| Mean temp of warmest quarter | 0.234 | **Drop** |  |  |  |  |  |  |  |  |  |  |  |
| Mean temp of coldest quarter | 0.136 | Retain | 24.94 | **Drop** |  |  |  |  |  |  |  |  |  |
| **Elevation variables** | | | | | | | | | | | | | |
| Elevation | 0.014 | Retain | 1.00 | Retain | 0.028 | Retain | 5.97 | Retain (lower AIC) | 0.579 | **Removed 2nd** |  |  |  |
| Slope | 0.570 | **Drop** |  |  |  |  |  |  |  |  |  |  |  |
| **Precipitation variables** | | | | | | | | | | | | | |
| Annual precipitation (prec) | 0.268 | **Drop** |  |  |  |  |  |  |  |  |  |  |  |
| Prec of wettest month | 0.095 | Retain | 1.06 | Retain | 0.071 | Retain | 1.58 | Retain | 0.293 | **Removed 4^th^** |  |  |  |
| Prec of driest month | 0.856 | **Drop** |  |  |  |  |  |  |  |  |  |  |  |
| Prec of wettest quarter | 0.378 | **Drop** |  |  |  |  |  |  |  |  |  |  |  |
| Prec of driest quarter | 0.612 | **Drop** |  |  |  |  |  |  |  |  |  |  |  |
| Prec of warmest quarter | 0.155 | Retain | 1.25 | Retain | 0.030 | Retain | 3.65 | Retain | 0.501 | **Removed 3^rd^** |  |  |  |
| Prec of coldest quarter | 0.148 | Retain | 1.18 | Retain | 0.016 | Retain | 6.86 | **Drop** (higher AIC) |  |  |  |  |  |
| **Soil composition** | | | | | | | | | | | | | |
| Soil texture | 0.479 | **Drop** |  |  |  |  |  |  |  |  |  |  |  |
| Soil pH | <0.001 | Retain | 1.00 | Retain | <0.001 | Retain | 3.53 | Retain | 0.051 | Retain | 0.001 | Retain | X |
| **Landcover/ vegetation** | | | | | | | | | | | | | |
| Landcover type | 0.212 | **Drop** |  |  |  |  |  |  |  |  |  |  |  |
| NDVI | 0.023 | Retain | 3.01 | Retain | 0.554 | **Drop** |  |  |  |  |  |  |  |
| EVI | 0.028 | Retain | 3.01 | Retain | 0.617 | **Drop** |  |  |  |  |  |  |  |

**References**

1. Basuni M, Muhi J, Othman N, Verweij JJ, Ahmad M, Miswan N, et al. A pentaplex real-time polymerase chain reaction assay for detection of four species of soil-transmitted helminths. Am J Trop Med Hyg. 2011;84(2):338-43. Epub 2011/02/05. doi: 10.4269/ajtmh.2011.10-0499. PubMed PMID: 21292911; PubMed Central PMCID: PMCPMC3029194.

2. Liu J, Gratz J, Amour C, Kibiki G, Becker S, Janaki L, et al. A laboratory-developed TaqMan Array Card for simultaneous detection of 19 enteropathogens. J Clin Microbiol. 2013;51(2):472-80. Epub 2012/11/24. doi: 10.1128/JCM.02658-12. PubMed PMID: 23175269; PubMed Central PMCID: PMCPMC3553916.

3. Zendejas-Heredia PA, Colella, V., Hii, S. F., Traub, R. J. Comparison of the egg recovery rates and limit of detection for soil-transmitted helminths using the Kato-Katz thick smear, faecal flotation and quantitative real-time PCR in human stool. PLoS Negl Trop Dis. 2021;15(5):e0009395. doi: <https://doi.org/10.1371/journal.pntd.0009395>.

4. Verweij JJ, Canales M, Polman K, Ziem J, Brienen EA, Polderman AM, et al. Molecular diagnosis of Strongyloides stercoralis in faecal samples using real-time PCR. Trans R Soc Trop Med Hyg. 2009;103(4):342-6. Epub 2009/02/07. doi: 10.1016/j.trstmh.2008.12.001. PubMed PMID: 19195671.

5. Lambert SB, Whiley DM, O'Neill NT, Andrews EC, Canavan FM, Bletchly C, et al. Comparing nose-throat swabs and nasopharyngeal aspirates collected from children with symptoms for respiratory virus identification using real-time polymerase chain reaction. Pediatrics. 2008;122(3):e615-20. Epub 2008/08/30. doi: 10.1542/peds.2008-0691. PubMed PMID: 18725388.

6. Verweij JJ, Brienen EA, Ziem J, Yelifari L, Polderman AM, Van Lieshout L. Simultaneous detection and quantification of Ancylostoma duodenale, Necator americanus, and Oesophagostomum bifurcum in fecal samples using multiplex real-time PCR. Am J Trop Med Hyg. 2007;77(4):685-90. Epub 2007/11/06. PubMed PMID: 17978072.

7. Hii SF, Senevirathna D, Llewellyn S, Inpankaew T, Odermatt P, Khieu V, et al. Development and Evaluation of a Multiplex Quantitative Real-Time Polymerase Chain Reaction for Hookworm Species in Human Stool. Am J Trop Med Hyg. 2018;99(5):1186-93. Epub 2018/09/19. doi: 10.4269/ajtmh.18-0276. PubMed PMID: 30226132; PubMed Central PMCID: PMCPMC6221243.
